# In Search of High-Yielding and Single-Compound-Yielding Plants: New Sources of Pharmaceutically Important Saponins from the Primulaceae Family

**DOI:** 10.3390/biom10030376

**Published:** 2020-02-29

**Authors:** Maciej Włodarczyk, Paweł Pasikowski, Kinga Osiewała, Aleksandra Frankiewicz, Andrzej Dryś, Michał Gleńsk

**Affiliations:** 1 Department of Pharmacognosy and Herbal Drugs, Wroclaw Medical University, Borowska 211 A, 50-556 Wrocław, Poland; 2 Mass Spectrometry Laboratory, Polish Center for Technology Development, Stabłowicka 147, 54-066 Wrocław, Poland; 3 Students Scientific Cooperation on Pharmacognosy, Wroclaw Medical University, Borowska 211 A, 50-556 Wrocław, Poland; 4 Department of Physical Chemistry and Biophysics, Wroclaw Medical University, Borowska 211 A, 50-556 Wrocław, Poland

**Keywords:** primrose root, *Primulae radix*, primulasaponin, sakurasosaponin, 13,28-epoxyoleanane saponins, *Primula*, *Dodecatheon*, *Auganthus*, biodiversity

## Abstract

So far, only a few primrose species have been analyzed regarding their saponin composition and content. Moreover, the roots of only two of them are defined by the European Union (EU) Pharmacopoeia monograph and commercially utilized by the pharmaceutical industry. Thus, this study intended to find some new sources of main triterpene saponins from *Primulae radix*, namely primulasaponins I and II together with the closely related sakurasosaponin. Using isolated standards, UHPLC-ESI-HRMS served to assess over 155 Primulaceae members qualitatively and quantitatively. Nine examples of plants accumulating over 5% of primulasaponin I in their roots were found. Among them, in one case, it was found as the almost sole secondary metabolite with the concentration of 15–20% (*Primula grandis* L.). A reasonable content of primulasaponin II was found to be typical for *Primula vulgaris* Huds. and *P. megaseifolia* Boiss. & Bal. The sakurasosaponin level was found in seven species to exceed 5%. The finding of new, single and rich sources of the abovementioned biomolecules among species that were never analyzed phytochemically is important for future research and economic benefit. The chemotaxonomic significance of the occurrence of these three saponins in Primulaceae is discussed.

## 1. Introduction

Primulaceae Batsch. is a large family covering many perennial and herbaceous plants. Members of this family are widely distributed in the Northern Hemisphere, particularly in the meadows and rocky valleys of the Alps, Apennines, Pyrenees, Himalayas and North American Cordillera but also in dry regions of Kazakhstan, Iran and Turkey. Today, some of the primroses are known exclusively from herbaria specimens while several exist as endangered endemics. Since the time of Charles Darwin and A. K. Bulley, primulaceous plants have been a point of interest for botanists and alpine enthusiasts as well as for the gardeners who help to maintain threatened species and discover new ones [1,2,3,4,5]. Thanks to them, over 200 Primulaceae-related species remain in the plant trade apart from decorative varieties. Due to these and the fact that most *Primula* species possess partly recognized chemical composition [6,7,8], they provide an interesting field for phytochemical research.

According to the European Pharmacopoeia, the official primrose root (*Primulae radix*) may be obtained from *Primula veris* L. or *P. elatior* (L.) Hill. and is well known as an efficient secretolytic and expectorant drug [9,10,11]. It was widely introduced to European academic medicine as a surrogate of the American Senega root during WWI. It is still popular as a component of simple and complex pharmaceutical formulations [9]. It has been proven that the main saponins and active components of the official drug are primulasaponins: I (=primulasaponin A, **PSI**) and II (**PSII**), sometimes mentioned as primula acids due to the acidic character of their glycone part [9,12].

**Figure 1:**
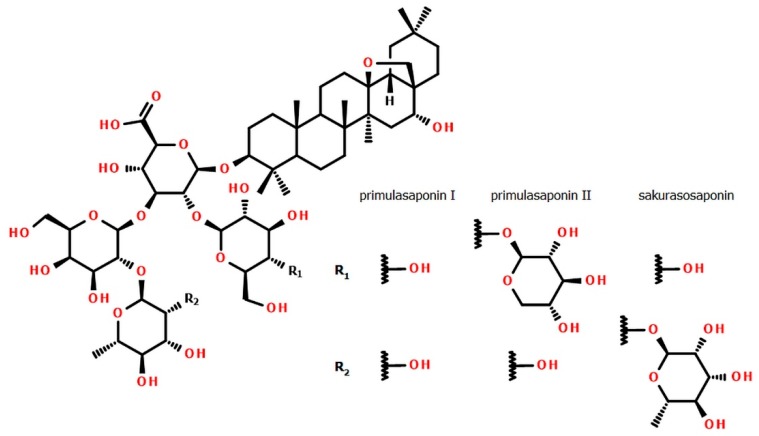
Structures of primulasaponins I, II and sakurasosaponin (**PSI**, **PSII** and **SSI**, respectively).

*Primula sieboldii* E. Morren is common in the Far East region (Japan, Amurland, Manchuria and Korea). The phytochemical value of this plant is connected with the triterpenoid biosynthesis yielding almost a single saponin, sakurasosaponin (**SSI**) [13], a close structural relative of **PSI** and **PSII** (Figure 1). However, ethnomedicinal applications of *P. sieboldii* to treat cough and bronchitis are not widely known [14].

Besides the well-known activities of saponins such as expectorant or vasoprotectant, an increasing number of researchers are interested in the evaluation of their promising positive interactions with, for example, chemotherapeutics [15,16,17]. Their usage in pharmacy, cosmetology and the food industry as natural and efficient emulsifiers or foaming agents is also desirable [18,19,20].

The glycosides and glycoside-esters of oleanane type are among the most widely distributed groups of saponins. Despite their wide occurrence, primulasaponins I, II and sakurasosaponin were previously not the object of any clinical study. However, they belong to 13,28-epoxyoleanane saponins that are of particular medical interest. Epoxidized oleanane derivatives were found to be active as enzyme inhibitors [21], anti-mycobacterial and anti-protozoan compounds [22,23] and selectively cytotoxic molecules [24,25]. It was demonstrated that the partially deglycosylated metabolites of long-chain 13,28-epoxyoleanane saponins also display antitumor activities at a level similar to their prodrug digested by intestinal flora [26].

Primulasaponins I and II, as well as sakurasosaponin, may serve as a readily available model compound for research (including structural modifications) in the mentioned areas of medicine. Particularly interesting are aldehydes such as ardisiacrispin B that can display, for example, cytotoxic effects in multi-factorial drug-resistant cancer cells [25,27]. The researchers know that the problem of the unavailability of large amounts of a single specialized metabolite is usually a hurdle in semi-synthesis. The number of unwanted by-products increases dramatically with a decrease in the purity of the substrate. Herewith, the use of single-metabolite yielding plants could be a strategy to work around this problem.

High-yielding sources of specialized natural compounds are not very frequent. The primary phytochemical education teaches that such compounds are usually found in concentrations that do not exceed 3–5% of the dry mass of plant material. Positive exceptions include quinine (>10% [28]) and some new sources of theobromine (>6%, *Camelia ptilophylla* [29]). Among phenylpropanoids, eugenol reaches 10–12% in cloves (at the level of >80% in essential oil). The highest yielding non-alkaloid substances usually occur in plants as complex mixtures (e.g., ~10% of tannins mixture in oak galls, ~6% of saponins mixture in horse-chestnut seeds). The LC-MS proven concentrations of single saponins in the Primulaceae family are usually about 2–6% [7,30].

The pharmaceutical industry prefers single, pure and well-defined biomolecules to serve as the standards. The most readily available compounds or the most active ones are used for the quality determination of raw herbal drugs as well as the reference for clinical studies. Single compounds can be conveniently evaluated, packed and dosed. On the other hand, the purification of natural compounds is usually bothersome and expensive, especially in the complex group of saponins. The lack of broad introduction of modern and rapid strategies for saponins’ determination results in perpetuating semi-quantitative, non-selective methods based on measurements of simple physicochemical properties (e.g., the new monograph included in European Pharmacopoeia (2.8.24) describes the “foam test” to evaluate the quality of saponin drugs [31]).

The objective of this study was to evaluate the distribution and average concentrations of the abovementioned saponins in commercially available Primulaceae species by the use of a cost-effective UHPLC-HRMS method [32]. The second target of this study was to find any species producing the main active compounds in significant quantities preferably as sole substances. The final goal was to identify primulas that could be further used as substitutes for cowslip and oxlip in medical use or utilized as a raw material to efficiently produce significant quantities of selected saponin compounds for commercial purposes or pharmacological deep-research needs.

## 2. Materials and Methods

### 2.1. Chemicals

LC-MS grade solvents were purchased from Merck (Darmstadt, Germany) and Sigma-Aldrich (St. Louis, MO, USA) while those of analytical grade were from Chempur (Piekary Śląskie, Poland) and POCh (Lublin, Poland).

### 2.2. Plant Material

The roots of the Primulaceae representatives were obtained from various botanical collections and nurseries. A total amount of 157 taxa were gathered. Of 111 *Primula* species and varieties, 60 belonged to *Aleuritia*, 11 to *Auganthus*, 30 to *Auriculastrum* and 10 to *Primula* subgenera. Additionally, the following primrose relatives were collected: 24 *Androsace* members together with 5 *Cortusa*, 2 *Dionysia*, 2 *Hottonia*, 2 *Lysimachia*, 2 *Omphalogramma*, 6 *Soldanella* and 3 *Vitaliana* taxa. Some were repeated in consecutive years. Plants were authenticated [1,2,3,4,33] and documented by photography by the author (M.W.). The plants were harvested in the late summer stage of growth except for lysimachias and hottonias (spring/summer). As a comparison, four trade samples of primrose root deposited in the collection of the Department of Pharmacognosy and Herbal Drugs were used.

The roots were carefully washed, separated from rhizomes and leaves and dried at room temperature in the shade. Vouchers of plant substances were deposited in the Department of Pharmacognosy and Herbal Drugs of Wroclaw Medical University. The precise list of all plant samples and their origin (donators) is combined in Appendix A Table A1.

### 2.3. Preparation of Samples

For HPLC evaluation, each sample was precisely weighed (100 mg), transferred into a sealed vial and extracted with 2 mL of 70% MeOH for 15 minutes in an ultrasonic bath (Bandelin, Berlin, Germany) at 25 °C and 50% of the power. 1 mL of each extract was filtered using 0.22 μm PTFE single-use syringe filters (Merck-Millipore, Darmstadt, Germany), diluted 100 times with acetonitrile/water mixture (1/1, *v*/*v*; LC-MS class) and stored at 4 °C before analysis [32].

### 2.4. Preparation of Standards

The standards of primulasaponin I and II were isolated from the commercial primrose root (complies with Pharmacopoeia requirements; Galke GmbH, Gittelde, Germany). The procedure was as follows: 50 g of the root was homogenized (A11; IKA, Königswinter, Germany) and extracted twice with 0.5 L of 70% methanol. The resulting extract was concentrated using a rotary evaporator (Büchi, Flawil, Switzerland) at 40 °C, diluted with water, applied on a Diaion HP-20 SPE column (Sigma-Aldrich, St. Louis, MO, USA) and eluted with water/methanol of an increasing gradient. Fractions eluted with 50% MeOH, containing a mixture of saponins (702 mg; 1.4% of starting material), were combined, concentrated and subjected to LC on silica (Merck, Darmstadt, Germany) in ethyl acetate/acetic acid/water (5/1/1, *v*/*v*/*v*) as the mobile phase [16]. Later, the eluates were collected according to their TLC profiles. For TLC analysis, the same solvent system was used as for LC on silica. Finally, the combined eluates were purified by solid-phase extraction (SPE) on the Chromabond C18 column (Macherey-Nagel, Düren, Germany) giving as a result pure saponins **PSI** (33 mg) and **PSII** (73 mg).

The standard of sakurasosaponin was isolated from commercial *P. sieboldii* roots (Kevock Garden, Lasswade, UK). 1.60 g of the powdered root was extracted by 1-day maceration with 70% methanol. The extract was diluted with water and subjected to SPE on the Chromabond C18 column. The fraction eluted with 70% methanol was collected, evaporated to dryness (60 mg) and finally purified by preparative TLC on an RP-18 W plate (Macherey-Nagel, Düren, Germany), giving as a result 25 mg of **SSI**.

The saponins were stored as powders at 4 °C before the NMR and HRMS analysis. An aliquot of isolated saponins was then subjected to HRMS, ^1^H- and ^13^C NMR spectroscopy, including 2D experiments and compared positively against assignments from the literature [12,13].

The stock solutions containing 2.0 mg/mL, 2.4 mg/mL and 2.2 mg/mL of **PSI**, **PSII** and **SSI** respectively were prepared in acetonitrile/water (1/1, *v*/*v*) and stored at 4 °C before the analytical use.

### 2.5. General NMR and HRMS Experimental Procedures

^1^H, ^13^C and 2D NMR spectra were obtained on a Bruker Avance 300 NMR spectrometer (Bruker BioSpin, Rheinstetten, Germany), operating at 300 MHz and 75 MHz respectively at 300 K, using standard pulse programs and methanol-*d4* (Armar AR, Döttingen, Switzerland) as well as DMSO-*d6* (Armar AR) as the solvent. HRMS measurements of isolated compounds were conducted on the ESI-qTOF Maxis instrument (Bruker Daltonics, Bremen, Germany) while for UHPLC-HRMS detection ESI-qTOF Compact (Bruker Daltonics) was used. The instruments were operated in negative mode and calibrated with the Tunemix mixture (Bruker Daltonics) with *m/z* standard deviation below 0.5 ppm. In the case of the UHPLC-MS measurements a calibration segment was introduced at the beginning of every single run. The mass accuracy of saponin standards was within 3 ppm. The analysis of the obtained mass spectra was carried out using Data Analysis and Quant Analysis (Bruker Daltonics) software. The main instrumental parameters were as follows: scan range 50–2200 *m/z*, nebulizer pressure 1.5 bar, dry gas (N_2_) 7.0 L/min, temperature 200 °C, capillary voltage 2.2 kV, ion energy 5 eV, collision energy 10 eV, low mass set at 200 *m/z*. The samples were dissolved in acetonitrile/water (1:1, *v*/*v*) containing 0.1% HCOOH.

### 2.6. Purity of Standards

The purity of isolated standards of saponins was ascertained using a ^1^H-qNMR method with maleic acid (Fluka, Buchs, Switzerland) as the internal standard with declared 99.94% content. Precisely weighed samples of each saponin were mixed with the precisely weighed standard and dissolved in 1 mL of methanol-*d4* (Armar AR) in two repetitions. 600 μL of each was used for analysis in a 5 mm tube on 700 MHz Bruker apparatus equipped with TXI CP (Bruker BioSpin). The analysis was conducted according to the procedure of the Polish Center for Technology Development, based on [34].

### 2.7. LC-MS Analytical Conditions

The Thermo Scientific UHPLC Ultimate 3000 apparatus (Thermo Fisher Scientific, Waltham, MA, USA) consisted of an LPG-3400RS quaternary pump with a vacuum degasser, a WPS-3000RS autosampler and a TCC-3000SD column oven. The ESI-qTOF Compact (Bruker Daltonics, Bremen, Germany) was connected as the MS detector. The separation was achieved on a Kinetex C-18 column (150 × 2.1 mm) of 2.6 μm particle size, core-shell type (Phenomenex, Torrance, CA, USA). The gradient elution system consisted of 0.1% HCOOH in water (mobile phase A) and 0.1% HCOOH in acetonitrile (mobile phase B). At the flow rate of 0.3 mL/min, the following elution program was used: 0→1 min (2%→30% B), 1→31 min (30%→60% B), 31→31.5 min (60%→100% B), 31.5→35.5 min (100% B). The column was equilibrated for 7 min before the next analysis. Blanks were added after each run to avoid any sample carryover. All analyses were carried out isothermally at 30 °C. The injection volume for samples and standard solutions was 5 μl. Each analysis was calibrated in the first segment of analysis and performed in duplicate. The method was as in Reference [32].

### 2.8. Validation of the Analytical Method

The UHPLC–MS assay was validated with respect to the specificity, linearity, precision, accuracy and stability.

#### 2.8.1. Specificity

Concentrations of the saponins were calculated using areas of peaks from EIC chromatograms extracted for 1103.564 ± 0.01 *m/z* (**PSI**, 15.67 min ± 0.50 min), 1235.606 ± 0.01 *m/z* (**PSII**, 14.07 min ± 0.50 min) and for 1249.622 ± 0.01 *m/z* (**SSI**, 15.10 min ± 0.50 min). The concentrations were measured in triplicate. Each sample was evaluated manually for the absence of closely eluting interferences (within ± 0.50 min of the nominal retention time) using the abovementioned extraction and UHPLC-MS conditions. Because every taxon possessed different patterns of interfering compounds, validation parameters were analyzed using standards.

#### 2.8.2. Linearity, Range and Limits of Analysis

The linearity was achieved by assaying a series of the mixed standard solution (**PS I**, **PS II**, **SSI**; consisting of 20 analytic points for each analyte; in a range of 0.050-250 µg/mL) in duplicate over three consecutive days. The efficient calibration equation to assure the calibration curve fit in the whole range of detector response as well as to include different saponin content in samples was proposed to be:

(1) $$y=\sqrt[{n}]{\frac{B^{n}\times x}{A-x}}$$

Using Statistica 12.5 software (Tulsa, OK, USA), the variable parameters of equation (n, A, B) together with coefficients of correlation (r) and coefficients of determination (r^2^) were calculated for each curve. The limits of detection (LOD) and quantification (LOQ) were evaluated by the signal-to-noise approach with the use of the lowest concentration. All results are given in Table 1.

#### 2.8.3. Precision, Accuracy and Stability

Precision, accuracy and stability were determined using standards at low, medium and high concentration levels (1, 10 and 100 µg/mL for all saponins, corresponding to 0.2%, 2.0% and 20% of root dry mass). All concentration levels were measured in triplicate. The precision and accuracy were tested once a day and repeated for three consecutive days. Intra- and interday precision were defined as the relative standard deviation (RSD), while the accuracy was determined by the relative error (RE %). The stability of analyzed saponins was assessed using standards stored at room temperature for 14 days in three repetitions. All results are gathered in Table 2.

### 2.9. Clustering of Saponin Concentrations

**Figure 2:**
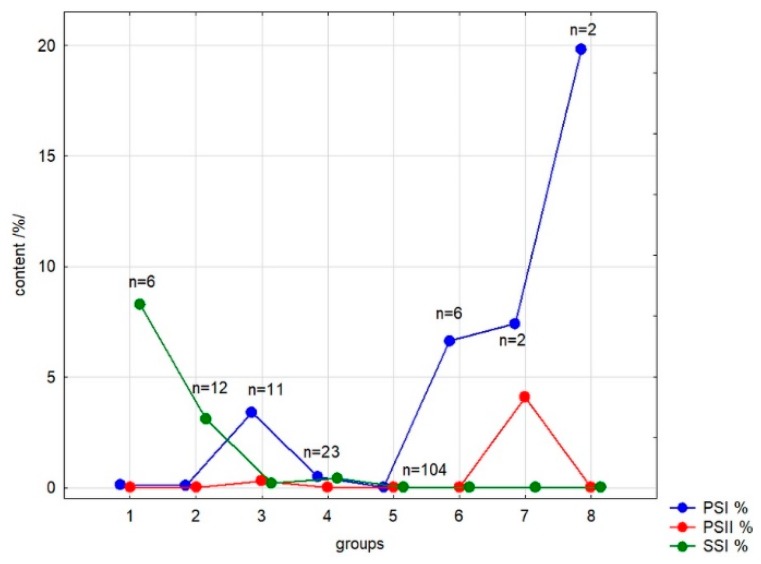
Visual representation of clustering analysis (CA) of analyzed Primulaceae taxa; based on the content of primulasaponins I, II and sakurasosaponin (**PSI**, **PSII** and **SSI**).

Using Statistica 12.5 software (Tulsa, OK, USA), clustering analysis (CA) was performed to group the samples according to the content of each saponin. The results are presented at Figure 2 and Table 3.

## 3. Results

### 3.1. Method Development

In our study, only species offered year by year in common trade in Europe were collected, trying to obtain at least 2–3 species from each available botanical section or subsection. Finally, 157 taxa were collected to perform the screening (collected in Table A1). The roots of the authenticated plants were sequentially dried, milled, extracted with 70% MeOH, filtered and diluted to obtain samples applicable for the assay. The universal extraction strategy was based on observations during the isolation process: SPE fractions eluted with 70% MeOH from C18 bed were the richest in isolated saponins. Ultrasound-assisted extraction (UAE) is a common strategy for the rapid extraction of both fresh and dry plant material. It was first-choice when small amounts of plant material were available.

Because the reference substances of all mentioned saponins were not simply available, the standards were isolated from pharmacopoeial primrose roots (**PSI** and **PSII**) and the roots of *Primula sieboldii* (**SSI**). Their structures (Figure 1) were elucidated based on the following HRMS and NMR experiments. High-resolution mass spectrum measured for the investigated creamy amorphous solids in negative ion mode revealed the following main peaks: for **PSI** [M–H]^−^ at 1103.5643 *m/z* (calculated for C_54_H_87_O_23_; 1103.5644), for **PSII** [M–H]^–^ at 1235.6054 *m/z* (calculated for C_59_H_95_O_27_; 1235.6066) and for **SSI** [M–H]^−^ at 1249.6241 *m/z* (calculated for C_60_H_97_O_27_ 1249.6223). The formulas generated for detected ions corresponded well to neutral molecules C_54_H_88_O_23_ (**PSI**), C_59_H_96_O_27_ (**PSII**) and C_60_H_98_O_27_ (**SSI**) and the MS/MS fragmentation of the glycone part was the additional proof. All HRMS and MS/MS spectra are attached to the Supplementary Figures S1 and S2. The detailed ^1^H and ^13^C NMR spectra, combined with COSY, HSQC and HMBC experiments, finally confirmed the identities of isolated compounds (Supplementary Figures S3–S5) with regard to the literature [12,13]. Table S1, summarizing NMR measurements, is attached to Supplementary Material. The purity level that is needed for the circumspect usage of isolated compounds in the quantitative analysis was calculated by qHNMR as 88.91% (**PSI**), 78.14% (**PSII**) and 91.89% (**SSI**).

Limited by the fact that the **PSI**, **PSII** and **SSI** saponins are not applicable for efficient and reliable HPLC-UV analysis due to the lack of conjugated double bonds except carbonyl groups, we used a previously developed simple UHPLC-ESI-MS analytical method for their precise quantitative evaluation [32]. On its basis, a qualitative method was proposed to rapidly estimate the concentrations. A non-standard curve (1) (Table 1) was fit to cover the response of the detector in a broad range of concentrations (0.050–250 µg/mL, relative to 0.01–50% of root dry mass). The correlation coefficients were all of 0.999. The LOQ values were between 19 and 23 ng/mL.

The full validation of the quantitative method was intentionally not performed at this level of research; however, some data are collected in Table 2 to supply the assay. Values of inter-day and intra-day precision are acceptable, while low accuracy at the lowest level suggested the measurement uncertainty and need to use higher concentrations. The standards seemed to be stable in two weeks at room temperature.

The MS detector was calibrated before each UHPLC analysis to guarantee accuracy. Samples were separated by blank analyses to exclude the possibility of overlapping. The low flow of chromatographic solvents together with the relatively fast gradient program resulted in eco-friendliness. The primarily considered faster way of HRMS quantitative analysis in the “direct injection mode” (without LC) was rejected because of the detection of ions isobaric to **PSI** and **SSI** in some of the first chromatograms. These ions were sufficiently separated from analytes and did not affect the area readings.

### 3.2. Distribution of Saponins

Figure 2 and Table 3 show the result of K-means clustering (CA). Table 4 shows in the form of a heatmap the average concentrations of analyzed saponins in roots of 157 Primulaceae members (> 165 samples). Only the exemplars with the saponin level exceeding 0.20% were presented with percentages (calculated from root dry mass) in the Appendix A, Table A2. The concentrations of **PSI**, **PSII** and **SSI** were under the LOD or the compounds were present in trace amounts in representatives of genera *Cortusa* L. (also considered as a part of the *Primula* L. [35]), *Dionysia* Fenzl., *Lysimachia* L., *Omphalogramma Franch.*, *Soldanella* L., *Vitaliana* Sesl. and in a part of *Androsace* L.

#### 3.2.1. Distribution of Primulasaponins

The presence of both **PSI** and **PSII** was established to be typical only for the genus *Primula* L. and subgenus *Primula* (0.1–6.2% and 0–3.3% respectively). **PSII** was not found in considerable amounts elsewhere. Moreover, the second compound was found in significant amounts, exceeding 2% in roots of only two species, *P. vulgaris* Huds. and *P. megaseifolia* Boiss. & Bal. Contrary to **PSII**, the saponin **PSI** was also present in *Primula* L., subgenus *Aleuritia* (Duby) Wendelbo, in sections *Crystallophlomis* (Rupr.) Federov (0.1–9.5%), *Davidii* Balf. f. (<1%), *Oreophlomis* (Rupr.) Federov (1.7–5.2%) and *Petiolares* Pax (about 1%). Among *Proliferae*-belonging taxa, only *P. japonica* reached the level of 1% of **PSI**. In members of subgenera *Auganthus* (Link) Wendelbo and *Auriculastrum* Schott only trace amounts of **PSI** were found except for *Primula parryi* A. Grey roots (~1%). *Primula grandis* L., a single representative of *Primula* L., section *Sredinskya* Stein was newly found to concentrate the highest amounts of **PSI** among all samples (15–20%). After this observation, a similar level of concentration was also found in its remarkably fleshy rhizomes.

Some years ago, the genus *Dodecatheon* L. was proposed to be included in *Primula* L. based on genetic analyses [36]. Our findings on **PSI** concentrations in 7 species from the *Dodecatheon* L. were generally similar to its relative subgenus *Auriculastrum*. However, two former *Dodecatheon* members—*P. conjugens* (Greene) Mast & Reveal and *P. jeffreyi* (van Houtte) Mast & Reveal—were quite abundant in **PSI** saponin (3.9% and 2.9% respectively).

#### 3.2.2. Distribution of Sakurasosaponin

Sakurasosaponin, primarily detected and described only in *P. sieboldii* E. Morren, was found to be typical for almost all of its analyzed relatives from the subgenus *Auganthus* (Link) Wendelbo (range of 1.1–9.1%). Its presence in the subgenus *Auriculastrum* Schott, section *Cuneifolia* Balf. f. (2.3–3.3%) and former genus *Dodecatheon* L. (0.7–8.6%) is noteworthy. Particularly high amounts of **SSI** were newly discovered in *P. forrestii* Balf. f. (~8%), *P. obconica* Hance and *P. takedana* Tatew. (both >8%). *P. jesoana* Miq was found to be the only species from the subgenus *Auganthus* in which sakurasosaponin was absent. Relatively small amounts of **SSI** were detected in both *Hottonia* specimens (0.6–2.3%) and several representatives of *Androsace* (0.1–1.3%).

#### 3.2.3. Results of Clustering Analysis

K-means clustering led to the separation of a dataset into eight groups of different numbers (Figure 2). The most numerous group 5 consists of samples containing no one of analyzed compounds at a concentration higher than 0.2%; the second numerous group 4 contains samples with poor concentrations of all three saponins. Group 2 collects the samples with medium amounts of the sakurasosaponin (mainly from subgenus *Auganthus*—section *Cortusoides* and subgenus *Auriculastrum*—sections *Cuneifolia* and *Dodecatheon*) while samples containing large amounts of this compound are gathered in group 1 (exemplars from subgenera *Auganthus*—*P. forrestii*, *P. obconica*, *P. takedana*—and *Auriculastrum*—*P. angustifolia*, *P. clevelandii* and *P. tetrandra*). Group 3 consists of samples with an medium content of primulasaponin I, groups 6 and 7—high content of this compound (in case of group 7, accompanied by significant amounts of primulasaponin II). Samples very rich in single primulasaponin I from subgenus *Primula*, section *Sredinskya* are in group 8. Ascriptions of samples to groups 1–3 and 6–8 are presented in Table 3.

## 4. Discussion

The lack of high-yielding or single-compound-yielding plant sources of active constituents is a common problem for institutions that develop standardization procedures of herbal drugs and those involved in deep research or clinical studies. In order to improve the quality and the value of medicinally important crops, several attempts are made in different fields. Some try to modify environmental conditions. Others try modern genetic manipulations. The recent trends in this area are also focused on the importance of soil microorganisms and mycorrhizal fungi [37,38]. The old strategy to increase the production of crucial secondary metabolites is to conduct organized selection of better-yielding cultivars, varieties or hybrids. The latter approach may still work successfully since 21st-century knowledge on medicinal plants is still rudimentary.

To find new and abundant sources of primulasaponins I and II and closely related sakurasosaponin, over 155 taxa of Primulaceae members were analyzed by the newly designed universal method. Most of them were not previously mentioned in the literature in the phytochemical context. Limited by the fact that the **PSI**, **PSII** and **SSI** saponins are not applicable for efficient HPLC-UV analysis, a simple UHPLC-ESI-MS analytical method was developed for this purpose. Previously UHPLC-APCI-MS, in comparison with UHPLC-ELSD, was used for **PSI** and **PSII** quantification in pharmacopoeial herbal drugs [7]. For assay purposes, the standards of both primulasaponins (**PSI** and **PSII**) were isolated from the pharmacopoeial primula root while sakurasosaponin (**SSI**) was isolated from the roots of *P. sieboldii* by LC and FC. The identity of compounds was confirmed by comparison of spectrometric and spectroscopic data (HRMS, MS/MS and NMR) with the literature [12,13], while their purity was determined by qHNMR.

According to the literature, the saponin content in *Primula* species may vary from 2% to 12% but the exact range of expectation is not defined in the monographs [9,10,11]. Our study shows that the roots of non-pharmacopoeial primroses may be an alternative source for **PSI** isolation, sometimes exceeding the level of 10%. Some of the selected species exhibit vigorous growth and are not very difficult to propagate (*P. conjugens*, *P. chionantha*, *P. macrophylla*, *P. vulgaris*). The other primarily promiscuous well-growing ones (e.g., from the section *Proliferae*) do not serve this purpose due to the lack of saponins mentioned above. *Primula grandis* L., a single representative of the section *Sredinskya*, should be considered as a particularly valuable source of this saponin. The reasons are as follows: almost complete exclusiveness of **PSI** in the saponin profile of this source (Figure S1), its unusual concentration exceeding 15–20% of both root and rhizome dry mass, together with significant growth and hardiness of this species.

Sakurasosaponin, which was previously reported only from *P. sieboldii*, appears to be typical for many *Auganthus* and *Dodecatheon* members. For example, *P. takedana* and *P. obconica* may yield reasonable amounts of this compound with almost no coexisting saponins. The structure of this compound (**SSI**) is quite similar to those of **PSI** and **PSII**. The above, together with the ethnomedicinal applications of *P. sieboldii*, should lead soon to the consideration of **SSI** as a substitute for **PSI** and **PSII** or their mixtures (in the form of extracts).

The information presented in the Figure 2 and Table 3 and Table 4 could be valuable as supplementary data for taxonomists analyzing phylogenetic relationships in this family [39,40]. Some previously underestimated species should be recognized as remarkably useful for the pharmaceutical industry.

Frequently, phytochemical studies are limited to a few species and use different methods. Here, by using a unified approach, a reasonable number of plant species was tested. The samples were supplied for seven consecutive years (2013-2019) to cover the maximal number of Primulaceae species available in commercial trade. Attempts were made to sufficiently cover the number of representatives in each section. It allowed us to select some notable cases. In the light of the presented results, ornamental hybrids and varieties of *Primula* species belonging to the subgenera *Primula* and *Auganthus* were selected to continue the systematic exploration. This sizeable comparative study is a good starting point for further research covering seasonal and environmental saponin variation as well as for establishing metabolomic relationships among primroses.

This work presents the first systematic study of three 13,28-epoxy-oleanane-type saponins (**PSI**, **PSII**, **SSI**) in the genus *Primula* by UHPLC-ESI–MS. Although the mentioned saponins were previously not a subject of any clinical study, the discovery of abundant and single-compound sources of primulasaponins and sakurasosaponin should be a milestone in studies on their activities. As pure substances, they can be used directly or for structural modifications. The area of semisynthesis of new enzyme inhibitors and antibacterial or anti-protozoan agents is fertile. One direction for the application of their derivatives is a study on their cytotoxicity or modulation of action of well-known cytotoxic or antimicrobial agents.

## Figures and Tables

**Table 1:** Parameters of calibration Equation (1), for primulasaponins I (**PSI**), II (**PSII**) and sakurasosaponin (**SSI**), together with values of r, r^2^, LOD and LOQ.

Standard	n ± SD	A ± SD	B ± SD	r	r^2^	LOD [ng/mL]	LOQ [ng/mL]
**PSI**	0.890 ± 0.072	4,574,451 ± 83,044	24.73 ± 1.26	0.9998	0.9996	6.7	20.3
**PSII**	0.776 ± 0.110	3,274,550 ± 100,794	23.41 ± 2.24	0.9994	0.9988	6.4	19.4
**SSI**	0.943 ± 0.110	3,951,182 ± 86,066	20.29 ± 1.62	0.9995	0.9990	7.4	22.5

**Table 2:** Validation parameters for UHPLC-MS assay for primulasaponins I (**PSI**), II (**PSII**) and sakurasosaponin (**SSI**).

**Standard**	**Repeatability (RSD)/(Intra-Day Precision)**			**Intermediate Precision (RSD)/(Inter-Day Precision)**		
**Level**	**1 µg/mL**	**10 µg/mL**	**100 µg/mL**	**1 µg/mL**	**10 µg/mL**	**100 µg/mL**
**PSI**	2.3	2.0	0.4	5.6	1.6	1.2
**PSII**	4.0	1.7	0.7	6.1	1.5	1.1
**SSI**	2.8	1.2	1.0	3.0	1.2	0.9
**Standard**	**Accuracy (%RE)**			**Stability (RSD)/(14 days)**		
**Level**	**1 µg/mL**	**10 µg/mL**	**100 µg/mL**	**1 µg/mL**	**10 µg/mL**	**100 µg/mL**
**PSI**	+21.2	+3.5	+0.1	3.2	3.3	2.6
**PSII**	+30.6	+5.5	−0.7	10.1	1.8	4.2
**SSI**	+15.4	+4.8	+0.4	6.8	2.2	1.4

**Table 3:** List of outstanding groups resulting from clustering analysis; corresponds with Figure 2.

Sample Acronym	Group	Sample Acronym	Group	Sample Acronym	Group	Sample Acronym	Group
PFOR_K_2014	1	HOIN_MO_2014	2	PCHS_K_2015	3	PCHI_K_2014	6
POBC_K_2014	1	PZAM_K_2014	2	PAUA_K_2015	3	PCHC_K_2015	6
PTAK_E_2014	1	PCOR_B_2013	2	PROG_B_2013	3	PLON_K_2014	6
PCLE_K_2016	1	PPOL_K_2015	2	PCJG_K_2014	3	PMAC_K_2015	6
PTET_K_2014	1	PSIE_K_2014	2	PJEF_K_2015	3	PWAR_K_2014	6
PANG_K_2016	1	PHEU_K_2015	2	PELA_K_2014	3	PR2	6
		PPAL_K_2014	2	PMEG_K_2015	3		
		PCUN_K_2016	2	PVRS_K_2014	3	PVUL_K_2014	7
		PCUH_K_2016	2	PR3	3	PR1	7
		PAUS_K_2014	2	PR4	3		
		PMEA_K_2014	2	PMAG_B_2013	3	PGRA_K_2014	8
		PPAU_K_2014	2			PGRA_K_2016	8

PR1-4—samples of commercial trade *Primulae radix*, declared to fulfill pharmacopoeial requirements. Background color corresponds with botanical classification: blue—sg. *Auganthus*, yellow—sg. *Auriculastrum*, sct. *Dodecatheon*, green—sg. *Aleuritia*, sct., *Crystallophlomis*, pink—sg. *Aleuritia*, sct. *Oreophlomis*, grey—sg. *Primula*, sct. *Primula*, brown—sg. *Primula*, sct. *Sredinskya*, transparent—others.

**Table 4:** A heatmap of average primulasaponins I and II and sakurasosaponin distribution in *Primulaceae* family. Highest concentration—red, lowest concentration—green.

Taxon	n	PSI	PSII	SSI	Taxon (Continuation)	n	PSI	PSII	SSI	Taxon (Continuation)	n	PSI	PSII	SSI
**genus *Primula* L., sg. *Aleuritia***					**genus *Primula* L., sg. *Auganthus***					**genus *Androsace* L.**				
sct. *Aleuritia*, ssct. *Aleuritia*	3				sct. *Bullatae*	2				sct. *Aizodium*	1			
sct. *Aleuritia*, ssct. *Algida*	2				sct. *Cortusoides*, ssct. *Cortusoides*	3				sct. *Andraspis*	1			
sct. *Armerina*	5				sct. *Cortusoides*, ssct. *Geraniifolia*	4				sct. *Aretia*, ssct. *Aretia*	3			
sct. *Capitatae*	2				sct. *Obconicolisteri*	1				sct. *Aretia*, ssct. *Dicranothrix*	3			
sct. *Crystalophlomis*	1				sct. *Reinii*	1				sct. *Chamaejasme*, ssct. *Hookerianae*	1			
sct. *Crystalophlomis*, ssct. *Crystalophlomis*	7				**genus *Primula* L., sg. *Auriculastrum***					sct. *Chamaejasme*, ssct. *Mucronifoliae*	3			
sct. *Crystalophlomis*, ssct. *Maximowiczii*	3				sct. *Amethystina*	1				sct. *Chamaejasme*, ssct. *Strigillosae*	2			
sct. *Davidii*	2				sct. *Auricula*, ssct. *Arthritica*	2				sct. *Chamaejasme*, ssct. *Sublanatae*	2			
sct. *Denticulata*	3				sct. *Auricula*, ssct. *Auricula*	1				sct. *Chamaejasme*, ssct. *Villosae*				
sct. *Minutissimae*	1				sct. *Auricula*, ssct. *Brevibracteatum*	3				series *Chamaejasmoidae*	3			
sct. *Muscarioides*	3				sct. *Auricula*, ssct. *Chamaecallis*	2				series *Euvillosae*	3			
sct. *Oreophlomis*	4				sct. *Auricula*, ssct. *Cyanaster*	1				sct. *Douglasia*	1			
sct. *Petiolares*, ssct. *Edgeworthii*	2				sct. *Auricula*, ssct. *Erythrodosum*	2				sct. *Pseudoprimula*	1			
sct. *Petiolares*, ssct. *Griffithii*	2				sct. *Auricula*, ssct. *Rhopsidium*	3								
sct. *Petiolares*, ssct. *Petiolares*	1				sct. *Auricula*, hybrids	2				**genus *Cortusa* L.**	6			
sct. *Petiolares*, ssct. *Sonchifolia*	1				sct. *Cuneifolia*	2				**genus *Dionysia* Fenzl.**	2			
sct. *Proliferae*	5				sct. *Dodecatheon*	8				**genus *Hottonia* L.**	2			
sct. *Pulchella*	3				sct. *Parryi*	3				**genus *Lysimachia* L.**	2			
sct. *Sikkimensis*	6				**genus *Primula* L., sg. *Primula***					**genus *Omphalogramma* (Franch.) Franch.**	2			
sct. *Soldanelloides*	2				sct. *Primula*	6				**genus *Soldanella* L.**	6			
sct. *Yunnanensis*	2				sct. *Sredinskya*	1				**genus *Vitaliana* Sesl.**	3			
**genus *Primula*, sg. *Sphondyllia***	2				sct. *Primula*, hybrids	1								

**^hb^**—herb was used instead of roots; sg.—subgenus, sct.—section, ssct.—subsection, ssp.—subspecies, var.—variety, n—number of analyzed taxa in genus, section or subsection.

## Supplementary Materials

The following are available online at https://www.mdpi.com/2218-273X/10/3/376/s1, Figure S1: UHPLC-HRMS chromatograms of compounds **PSI**, **PSII** and **SSI** (EIC, negative mode) together with chromatograms of selected high-yielding primroses (TIC, negative mode). Figure S2: HRMS spectra and MS/MS fragmentation of compounds **PSI**, **PSII** and **SSI**. Figure S3: 1D and 2D NMR spectra of **PSI**. Figure S4: 1D and 2D NMR spectra of **PSII**. Figure S5: 1D and 2D NMR spectra of **SSI**. Figure S6: Purity determination protocol of compounds **PSI**, **PSII** and **SSI**. Table S1: ^1^H and ^13^C NMR data of **PSI**, **PSII** and **SSI**.

## List of Abbreviations

[M–H]^−^	Pseudomolecular Ion (deprotonated molecule)
2D	Two Dimensional (experiment in NMR)
*A.*	*Androsace*
APCI	Atmospheric-Pressure Chemical Ionization
COSY	^1^H-^1^H Correlated Spectroscopy (experiment in NMR)
ELSD	Evaporative Light Scattering Detector
ESI	Electrospray Ionization
EU	European Union
*H.*	*Hottonia*
HCOOH	Formic Acid
HMBC	Heteronuclear Multiple Bond Correlation (experiment in NMR)
HRMS	High-Resolution Mass Spectrometry
HSQC	Heteronuclear Single Quantum Correlation (experiment in NMR)
LOD	Limit of Detection
LOQ	Limit of Quantification
LC	Liquid Chromatography
LC-MS	Liquid Chromatography coupled with Mass Spectrometry Detector
LC-UV	Liquid Chromatography coupled with Ultraviolet Absorbance Detector
MeOH	Methanol
MS/MS	Tandem Mass Spectrometry
NMR	Nuclear Magnetic Resonance (Spectroscopy)
*P.*	*Primula*
PSI	Primulasaponin I
PSII	Primulasaponin II
qHNMR	Quantitative Proton Nuclear Magnetic Resonance (Spectroscopy)
r	Coefficient of Correlation
r^2^	Coefficient of Determination
RE	Relative Error
RSD	Relative Standard Deviation
sct.	Section
SD	Standard Deviation
sg.	Subgenus
SPE	Solid-Phase Extraction
ssct.	Subsection
ssp.	Subspecies
SSI	Sakurasosaponin
TLC	Thin-Layer Chromatography
UAE	Ultrasound-Assisted Extraction
UHPLC	Ultra-High-Performance Liquid Chromatography
var.	variety

## Appendix A

**Table A1:** List of Primulaceae species used for screening in this study.

**Taxon (a)**	**Acronym**	**Taxon (b)**	**Acronym**
**genus *Primula* L.**		sg. *Aleuritia*, sct. *Minutissimae*	
sg. *Aleuritia*, sct. *Aleuritia*, ssct. *Aleuritia*		*Primula primulina*	PPRI_P_2014
*Primula halleri*	PHAL_P_2013, PHAL_K_2016	sg. *Aleuritia*, sct. *Muscarioides*	
*Primula scandinavica*	PSCA_B_2013	*Primula concholoba*	PCON_K_2014
*Primula scotica*	PSCO_K_2016	*Primula muscarioides*	PMUS_P_2013
sg. *Aleuritia*, sct. *Aleuritia*, ssct. *Algida*		*Primula vialii*	PVIA_B_2013
*Primula algida*	PALG_K_2014	sg. *Aleuritia*, sct. *Oreophlomis*	
*Primula darialica*	PDAR_K_2014	*Primula auriculata*	PAUA_K_2015
sg. *Aleuritia*, sct. *Armerina*		*Primula luteola*	PLUT_K_2015
*Primula fasciculata*	PFAS_K_2014	*Primula rosea* ‘Gigas’	PROG_B_2013
*Primula involucrata*	PINV_P_2015	*Primula warschenewskiana*	PWAR_K_2014
*Primula munroi* ssp. *yargongensis* (->*P. involucrata* in [1])	PMUY_P_2015	sg. *Aleuritia*, sct. *Petiolares*, ssct. *Edgeworthii*	
*Primula yargongensis* (->*P. involucrata* in [1])	PYAR_P_2015	*Primula moupinensis*	PMOU_GP_2014
*Primula zambalensis*	PZAM_K_2014	*Primula moupinensis* ssp. *barkamensis*	PMOB_K_2015
sg. *Aleuritia*, sct. *Capitatae*		sg. *Aleuritia*, sct. *Petiolares*, ssct. *Griffithii*	
*Primula capitata* ssp. *mooreana*	PCAM_B_2013	*Primula calderiana* ssp. *calderiana*	PCAL_K_2014
*Primula glomerata*	PGLO_K_2014	*Primula tanneri* ssp. *nepalensis*	PTNN_K_2014
sg. *Aleuritia*, sct. *Crystalophlomis*		sg. *Aleuritia*, sct. *Petiolares*, ssct. *Petiolares*	
*Primula purdomii*	PPUR_P_2014	*Primula boothi* var. *repens*	PBOR_K_2014
sg. *Aleuritia*, sct. *Crystalophlomis*, ssct. *Crystalophlomis*		sg. *Aleuritia*, sct. *Petiolares*, ssct. *Sonchifolia*	
*Primula chionantha*	PCHI_K_2014	*Primula sonchifolia* ssp. *sonchifolia*	PSON_K_2014
*Primula chionantha* ssp. *chionantha*	PCHC_K_2015	sg. *Aleuritia*, sct. *Proliferae*	
*Primula chionantha* ssp. *sinopurpurea*	PCHS_K_2015	*Primula beesiana*	PBES_B_2013
*Primula graminifolia* (->*P. chionantha* in [1])	PGRM_P_2015	*Primula bulleyana*	PBUL_B_2013
*Primula longipetiolata*	PLON_K_2014	*Primula japonica*	PJAP_B_2013
*Primula macrophylla*	PMAC_K_2015	*Primula prolifera*	PPRO_K_2015
*Primula orbicularis*	PORB_K_2015, PORB_P_2015	*Primula wilsonii* var. *anisodora*	PWIA_K_2015
sg. *Aleuritia*, sct. *Crystalophlomis*, ssct. *Maximowiczii*		sg. *Aleuritia*, sct. *Pulchella*	
*Primula maximowiczii* var. *maximowiczii*	PMAX_K_2014	*Primula pulchella*	PPUL_K_2015, PPUL_P_2015
*Primula tangutica*	PTAN_P_2013	*Primula sharmae*	PSHA_K_2016
*Primula woodwardii*	PWOD_P_2013, PWOD_P_2014	*Primula stenocalyx*	PSTE_P_2013, PSTE_P_2014
sg. *Aleuritia*, sct. *Davidii*		sg. *Aleuritia*, sct. *Sikkimensis*	
*Primula bergenioides*	PBER_K_2015	*Primula firmipes*	PFIR_K_2014
*Primula ovaliifolia*	POVA_K_2015	*Primula florindae*	PFLO_B_2013
sg. *Aleuritia*, sct. *Denticulata*		*Primula florindae*, red flowered	PFLR_K_2015
*Primula denticulata* ‘Alba’	PDEA_B_2013	*Primula ioessa*	PIOE_P_2013
*Primula denticulata*	PDEN_K_2016	*Primula sikkimensis*	PSIK_K_2014
*Primula monticola*	PMON_K_2014, PMON_K_2015	*Primula waltonii*	PWAL_K_2014
**Taxon (c)**	**Acronym**	**Taxon (d)**	**Acronym**
sg. *Aleuritia*, sct. *Soldanelloides*		sg. *Auriculastrum*, sct. *Auricula*, ssct. *Erythrodosum*	
*Primula reidii*	PREI_K_2016	*Primula daoensis*	PDAO_K_2014
*Primula reidii* var. *williamsii* ‘Alba’	PREW_F_2015	*Primula hirsuta*	PHIR_P_2013
sg. *Aleuritia*, sct. *Yunnanensis*		sg. *Auriculastrum*, sct. *Auricula*, ssct. *Rhopsidium*	
*Primula florida* (*P. blinii*)	PBLI_P_2013, PBLI_P_2014	*Primula allionii*	PALL_F_2015
*Primula rupicola*	PRUP_K_2016	*Primula integrifolia*	PINT_P_2013
sg. *Auganthus*, sct. *Bullatae*		*Primula tyrolensis*	PTYR_TK_2015
*Primula bullata* var. *rufa*	PBUR_K_2014	sg. *Auriculastrum*, sct. *Auricula*, hybrids	
*Primula forrestii*	PFOR_K_2014	*Primula venusta*	PVEN_B_2013
sg. *Auganthus*, sct. *Cortusoides*, ssct. *Cortusoides*		*Primula allioni* × *Primula villosa*	PXAV_P_2013
*Primula cortusoides*	PCOR_B_2013	sg. *Auriculastrum*, sct. *Cuneifolia*	
*Primula polyneura*	PPOL_K_2015	*Primula cuneifolia*	PCUN_K_2016
*Primula sieboldii*	PSIE_K_2014	*Primula cuneifolia* ssp. *heterodonta*	PCUH_K_2016
sg. *Auganthus*, sct. *Cortusoides*, ssct. *Geraniifolia*		sg. *Auriculastrum*, sct. *Dodecatheon*	
*Primula heucheriifolia*	PHEU_K_2015	*Primula austrofrigida*	PAUS_K_2014
*Primula jesoana*	PJES_K_2014	*Primula clevelandii*	PCLE_K_2016
*Primula kisoana*	PKIS_K_2015	*Primula conjugens*	PCJG_K_2014
*Primula palmata*	PPAL_K_2014	*Primula jeffreyi*	PJEF_K_2015, PJEF_K_2016
sg. *Auganthus*, sct. *Obconicolisteri*		*Primula latiloba*	PLLB_K_2014
*Primula obconica*	POBC_K_2014	*Primula meadia*	PMEA_K_2014
sg. *Auganthus*, sct. *Reinii*		*Primula pauciflora*	PPAU_K_2014
*Primula takedana*	PTAK_E_2014	*Primula tetrandra*	PTET_K_2014
sg. *Auriculastrum*, sct. *Amethystina*		sg. *Auriculastrum*, sct. *Parryi*	
*Primula amethystina* var. *brevifolia*	PAMB_K_2015	*Primula angustifolia*	PANG_K_2016
sg. *Auriculastrum*, sct. *Auricula*, ssct. *Arthritica*		*Primula parryi*	PPAR_K_2014
*Primula glaucescens* ssp. *longobarda*	PGLL_P_2013, PGLL_P_2014	*Primula rusbyi*	PRUS_K_2014
*Primula spectabilis*	PSPE_P_2013	sg. *Primula*, sct. *Primula*	
sg. *Auriculastrum*, sct. *Auricula*, ssct. *Auricula*		*Primula elatior*	PELA_K_2014
*Primula auricula*	PAUR_B_2013	*Primula elatior* var. *amoena*	PAMO_P_2014
sg. *Auriculastrum*, sct. *Auricula*, ssct. *Brevibracteatum*		*Primula juliae*	PJUL_B_2013
*Primula carniolica*	PCAR_K_2014, PCAR_K_2015	*Primula megaseifolia*	PMEG_K_2015
*Primula latifolia* ‘Alba’	PLAA_P_2013	*Primula veris* (syn. *P. officinalis*)	PVRS_K_2014
*Primula marginata*	PMGN_P_2013	*Primula vulgaris*	PVUL_K_2014
sg. *Auriculastrum*, sct. *Auricula*, ssct. *Chamaecallis*		sg. *Primula*, sct. *Sredinskya*	
*Primula minima*	PMIN_I_2014	*Primula grandis*	PGRA_K_2014, PGRA_K_2016
*Primula minima* f. *niveum*	PNIV_P_2014	sg. *Primula*, hybrids	
sg. *Auriculastrum*, sct. *Auricula*, ssct. *Cyanaster*		*Primula margotae* ‘Garryarde Guinevere’	PMAG_B_2013
*Primula glutinosa*	PGLU_P_2013	sg. *Sphondyllia*	
		*Primula* × *kewensis*	PXKE_K_2016
		*Primula verticillata*	PVRT_K_2014
**Taxon (e)**	**Acronym**	**Taxon (f)**	**Acronym**
**genus *Androsace* L.**		sct. *Aizodium*	
sct. *Andraspis*		*Androsace bulleyana*	ABUL_F_2015
*Androsace albana*	AALB_K_2019	**genus *Cortusa* L.**	
sct. *Aretia*, ssct. *Aretia*		*Cortusa matthioli*	CMAT_P_2013
*Androsace cylindrica*	ACYL_K_2015	*Cortusa matthioli* ssp. *matthioli*	CMAT_K_2015
*Androsace lehmannii*	ALEH_P_2014	*Cortusa matthioli* ssp. *caucasica*	CCAU_K_2015, CCAU_K_2016
*Androsace mathildae*	AMTH_TK_2015	*Cortusa matthioli* ssp. *sachalinensis*	CSAC_P_2013, CSAC_K_2015
sct. *Aretia*, ssct. *Dicranothrix*		*Cortusa matthioli* ssp. *turkestanica*	CTUR_P_2013
*Androsace lacteal*	ALAC_TK_2015	**genus *Dionysia* Fenzl.**	
*Androsace laggeri (= A. carnea* var*. laggeri)*	ACAL_P_2014	*Dionysia khatamii*	DKHA_F_2015
*Androsace obtusifolia*	AOBT_P_2014	*Dionysia zschummelii*	DZSH_F_2015
sct. *Chamaejasme*, ssct. *Hookerianae*		**genus *Hottonia* L.**	
*Androsace limprichtii*	ALIM_P_2014	*Hottonia inflata* ^hb^	HOIN_MO_2014
sct. *Chamaejasme*, ssct. *Mucronifoliae*		*Hottonia palustris* ^hb^	HOPA_PG_2014
*Androsace mariae* var. *tibetica*	AMAT_P_2014	**genus *Lysimachia* L.**	
*Androsace mucronifolia*	AMUC_TK_2015	*Lysimachia nummularia* ‘Gold’	LYNG_PG_2014
*Androsace sempervivoides*	ASPV_B_2015	*Lysimachia thyrsiflora*	LYTH_MO_2014
sct. *Chamaejasme*, ssct. *Strigillosae*		**genus *Omphalogramma* (Franch.) Franch.**	
*Androsace spinulifera*	ASPI_P_2015	*Omphalogramma delavayi*	ODEL_PO_2019
*Androsace strigillosa*	ASTR_P_2014	*Omphalogramma tibeticum*	OTIB_K_2019
sct. *Chamaejasme*, ssct. *Sublanatae*		**genus *Soldanella* L.**	
*Androsace adenocephala*	AADE_F_2015	sct. *Crateriflorae*	
*Androsace nortonii*	ANOR_P_2014	*Soldanella alpina*	SALP_K_2016
sct. *Chamaejasme*, ssct. *Villosae*, series *Chamaejasmoidae*		*Soldanella carpatica*	SCAR_K_2014
*Androsace brachystegia*	ABRA_P_2014	*Soldanella cyanaster*	SCYA_K_2014
*Androsace chamaejasme* ssp. *carinata*	ACHC_P_2014	*Soldanella dimoniei*	SDIM_K_2014
*Androsace zambalensis*	AZAM_P_2014	*Soldanella villosa*	SVIL_K_2014
sct. *Chamaejasme*, ssct. *Villosae*, series *Euvillosae*		sct. *Tubiflorae*	
*Androsace dasyphylla*	ADAS_P_2014	*Soldanella minima*	SMIN_F_2015, SMIN_TK_2015
*Androsace robusta* ssp. *purpurea*	AROP_P_2014	**genus *Vitaliana* Sesl.**	
*Androsace sarmentosa*	ASAR_K_2015	*Vitaliana primuliflora*	VPRI_B_2015
sct. *Pseudoprimula*		*Vitaliana primuliflora* ssp. *assoana*	VPAS_TK_2015
*Androsace elatior*	AELA_F_2015	*Vitaliana primuliflora* ssp. *praetutiana*	VPPR_B_2015, VPPR_B_2017
sct. *Douglasia*			
*Androsace montana* (= *Douglasia montana*)	AMON_F_2015		

**^hb^**—herb was used instead of roots; sg.—subgenus, sct.—section, ssct.—subsection, ssp.—subspecies, var.—variety. The meaning of acronyms used to avoid long plant names in storage and during research is as follows: First letter—genus, three following letters—taxon (species; optionally together with variety), next separated letter or two—donator abbreviation and year of collection in the end. Donators abbreviations: B—Bergenia, Nursery, Paweł Weinar, Kokotów, Poland; E—Edrom, Nursery, Terry and Cath Hunt, Coldingham, UK; F—Floralpin, Nursery, Frank Schmidt, Waldenbuch, Germany; G—Private Collection, Gunhild and Thorkild Poulsen, Aldershvile, Denmark; I—Alpine Garden Mt. Patscherkofel, Institute of Botany, Innsbruck University, Peter Daniel Schlorhaufer, Innsbruck, Austria; K—Kevock Garden, Nursery, Stella and David Rankin, Lasswade, UK; MO—Mayla Ogrody, Nursery, Dawid Stefaniuk, Siedlakowice, Poland; P—Josef and Bohumila Plocar, Nursery, Švihov, Czech Republic; PG—Planta Garden, Krzysztof Sternal, Dobra, Poland; PO—Pottertons, Nursery, Jackie and Robert Potterton, Nettleton, UK; TK—Private Collection, Tomasz Kubala, Poland.

Table A2 Content of primulasaponin I, II and sakurasosaponin (**PSI**, **PSII** and **SSI**, relatively) in roots of selected Primulaceae species.

**Table:**

Taxon (a)	Average % of Dry Mass				Taxon (b)	Average % of Dry Mass				Taxon (c)	Average % of Dry Mass		
	PSI	PSII	SSI			PSI	PSII	SSI			PSI	PSII	SSI
**genus *Primula***					sg. *Auganthus*, sct. *Bullatae*					sg. *Auriculastrum*, sct. *Cuneifolia*			
sg. *Aleuritia*, sct. *Armerina*					*P. bullata* var. *rufa*	nd	nd	1.07		*P. cuneifolia*	Nd	nd	2.31
*P. zambalensis*	t	nd	2.97		*P. forrestii*	t	nd	8.00		*P. cuneifolia* ssp. *heterodonta*	Nd	nd	3.28
sg. *Aleuritia*, sct. *Crystallophlomis*					sg. *Auganthus*, sct. *Cortusoides*					sg. *Auriculastrum*, sct. *Dodecatheon*			
*P. chionantha*	5.57	nd	nd		*P. cortusoides*	nd	nd	4.37		*P. austrofrigida*	T	nd	2.62
*P. chionantha* ssp. *chionantha*	6.35	nd	nd		*P. heucherifolia*	t	nd	2.24		*P. clevelandii*	t	nd	8.59
*P. chionantha* ssp. *sinopurpurea*	4.31	nd	nd		*P. kisoana*	t	nd	1.68		*P. conjugens*	3.86	nd	1.26
*P. graminifolia*	1.22	nd	nd		*P. palmata*	t	nd	2.13		*P. jeffreyi*	2.85	nd	1.00
*P. longipetiolata*	9.48	nd	nd		*P. polyneura*	t	nd	2.22		*P. latiloba*	t	nd	0.77
*P. macrophylla*	7.06	nd	nd		*P. sieboldii*	t	nd	4.20		*P. meadia*	t	nd	3.56
*P. orbicularis*	0.60	nd	nd		sg. *Auganthus*, sct. *Obconicolisteri*					*P. pauciflora*	t	nd	5.08
*P. purdomii*	t	nd	nd		*P. obconica*	t	nd	8.59		*P. tetrandra*	t	nd	6.90
sg. *Aleuritia*, sct. *Davidii*					sg. *Auganthus*, sct. *Reinii*					sg. *Auriculastrum*, sct. *Parryi*			
*P. bergenioides*	0.73	nd	nd		*P. takedana*	t	nd	9.09		*P. angustifolia*	0.35	nd	8.60
*P. ovaliifolia*	t	nd	nd		sg. *Auriculastrum*, sct. *Auricula*					*P. parryi*	1.13	nd	0.44
sg. *Aleuritia*, sct. *Oreophlomis*					*P. allionii*	t	nd	nd		*P. rusbyi*	t	nd	0.85
*P. auriculata*	3.24	nd	nd		*P. allionii* × *P. villosa*	t	nd	nd		sg. *Primula*, sct. *Primula*			
*P. luteola*	1.75	nd	nd		*P. auricula*	t	nd	nd		*P. elatior* var. *amoena*	t	nd	nd
*P. rosea ‘Gigas’*	4.06	nd	nd		*P. carniolica*	t	nd	nd		*P. elatior*	3.30	nd	nd
*P. warschenewskiana*	5.21	nd	nd		*P. glaucescens* ssp. *longobarda*	t	nd	0.48		*P. elatior* ^rhiz^	4.63	nd	nd
sg. *Aleuritia*, sct. *Petiolares*					*P. glutinosa*	t	nd	nd		*P. juliae*	0.79	t	nd
*P. boothi* var. *repens*	1.04	nd	nd		*P. hirsuta*	t	nd	nd		*P. juliae* ^rhiz^	0.21	t	nd
*P. moupinensis*	t	nd	nd		*P. integrifolia*	t	nd	nd		*P. megaseifolia*	3.24	2.65	nd
*P. moupinensis* ssp. *barkamensis*	1.17	nd	nd		*P. latifolia ‘Alba’*	t	nd	nd		*P. veris* (syn. *P. officinalis*)	2.84	t	nd
*P. sonchifolia* ssp. *sonchifolia*	0.67	nd	nd		*P. marginata*	t	nd	nd		*P. vulgaris*	6.23	3.26	nd
sg. *Aleuritia*, sct. *Proliferae*					*P. minima*	t	nd	nd		*P. vulgaris* ^rhiz^	3.30	2.27	nd
*P. besiana*	t	nd	nd		*P. minima* f. *niveum*	t	nd	nd		sg. *Primula*, sct. *Sredinskya*			
*P. japonica ‘Alba’*	1.05	nd	nd		*P. tyrolensis*	t	nd	nd		*P. grandis* (PGRA_K_2014)	23.88	nd	nd
*P. prolifera*	t	nd	nd		*P. venusta*	t	nd	nd		*P. grandis* (PGRA_K_2016)	15.72	nd	nd
sg. *Aleuritia*, sct. *Pulchella*										*P. grandis* ^rhiz^ (PGRA_K_2016)	14.57	nd	nd
*P. pulchella*	t	nd	t

**Table:**

Taxon (d)	Average % of Dry Mass		
	PSI	PSII	SSI
sg. *Primula*, hybrids			
*P. margotae ‘GarryardeGuinevere’*	4.80	0.32	nd
*P. margotae* ^rhiz^	2.21	0.23	nd
sg. *Primula*, trade samples			
*Primulae radix* 1 (PR1)	8.60	4.90	nd
*Primulae radix* 2 (PR2)	6.16	t	nd
*Primulae radix* 3 (PR3)	2.66	t	nd
*Primulae radix* 4 (PR4)	2.18	t	nd
sg. *Sphondylia*, hybrids			
*P. × kewensis*	0.33	nd	nd
**genus *Androsace***			
sct. *Aretia*			
*A. lehmanii*	nd	nd	1.12
sct. *Chamaejasme*			
*A. adenocephala*	nd	nd	t
*A. brachystegia*	nd	nd	t
*A. chamaejasme* ssp. *carinata*	nd	nd	t
*A. limprichtii*	nd	nd	0.36
*A. robusta* ssp. *purpurea*	nd	nd	0.76
*A. sarmentosa*	nd	nd	1.28
*A. strigillosa*	t	nd	t
**genus *Hottonia***			
*H. inflata* ^hb^	0.36	nd	2.27
*H. palustris* ^hb^	0.31	nd	0.62

**^hb^**—herb or **^rhiz^**—rhizome was used instead of roots; sg.—subgenus, sct.—section, ssp.—subspecies, var.—variety, nd—below LOD, t—between LOQ and 0.2%. Values are averages of triplicates. Standard deviations lower than 5% in each case.
