# Supplementary material for: In Search of High-Yielding and Single-Compound-Yielding Plants: New Sources of Pharmaceutically Important Saponins from the Primulaceae Family
Source: Biomolecules. 2020 Feb 29;10(3):376. doi: 10.3390/biom10030376 (PMC7175136; doi:10.3390/biom10030376)
Supplement: Supplementary file 1 [file biomolecules-10-00376-s001.pdf]

33Communication

In Search of High-Yielding and Single-Compound-Yielding Plants: New Sources of Pharmaceutically Important Saponins from the Primulaceae Family

Supplementary Materials

Maciej Włodarczyk [1,*], Paweł Pasikowski [2], Kinga Osiewała [3], Aleksandra Frankiewicz [3], Andrzej Dryś [4], Michał Gleńsk [1]

1 Department of Pharmacognosy and Herbal Drugs, Wroclaw Medical University, Borowska 211 A, 50-556 Wrocław, Poland
2 Mass Spectrometry Laboratory, Polish Center for Technology Development, Stabłowicka 147, 54-066 Wrocław, Poland
3 Students Scientific Cooperation on Pharmacognosy, Wroclaw Medical University, Borowska 211 A, 50-556 Wrocław, Poland
4 Department of Physical Chemistry and Biophysics, Wroclaw Medical University, Borowska 211 A, 50-556 Wrocław, Poland
* Correspondence: maciej.wlodarczyk@umed.wroc.pl; Tel.: +48-71-78-40-223 (M.W.)

Figure S1: UHPLC-HRMS chromatograms of compounds **PSI**, **PSII** and **SSI** (EIC, negative mode) together with chromatograms of selected high-yielding primroses (BPC, negative mode). Abbreviations of samples in Table A1. Conditions in chapter 2.6.

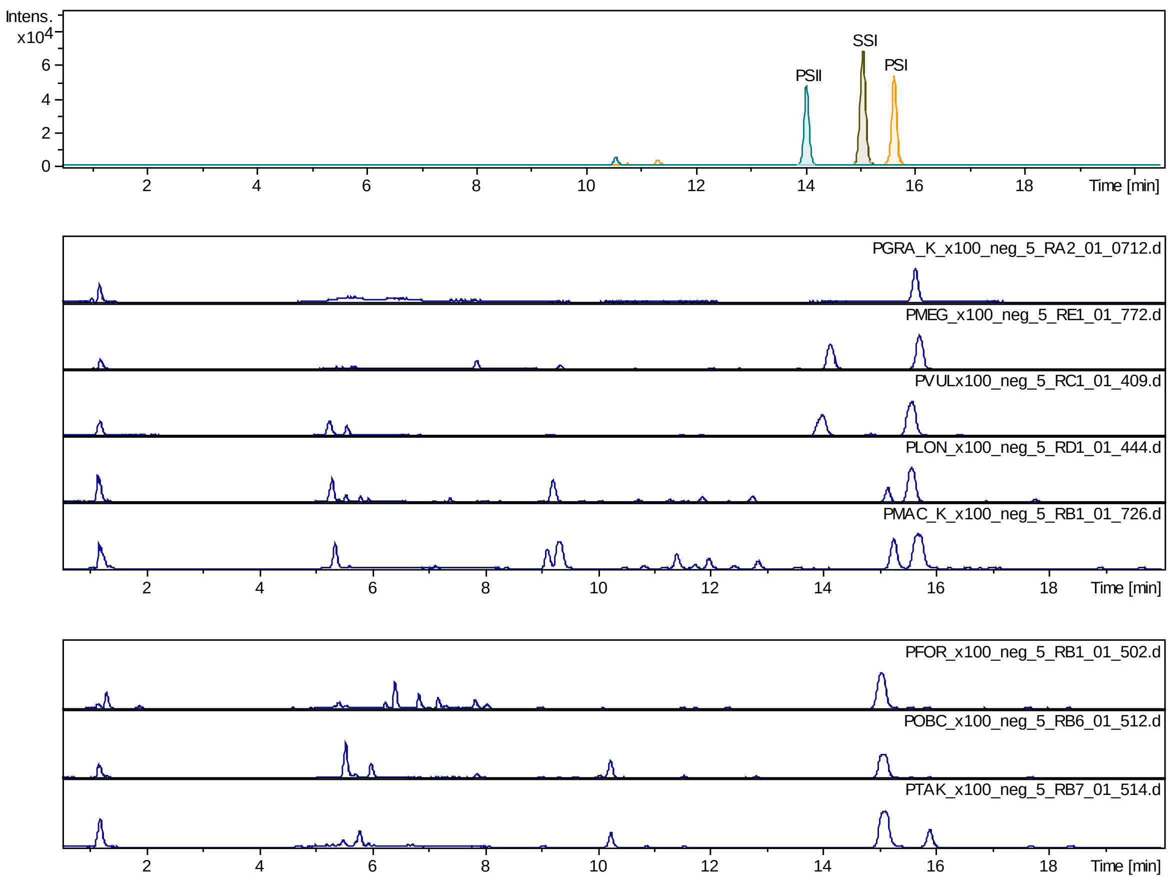

Figure S2: HRMS spectra and MS/MS fragmentation of compounds **PSI**, **PSII** and **SSI**.

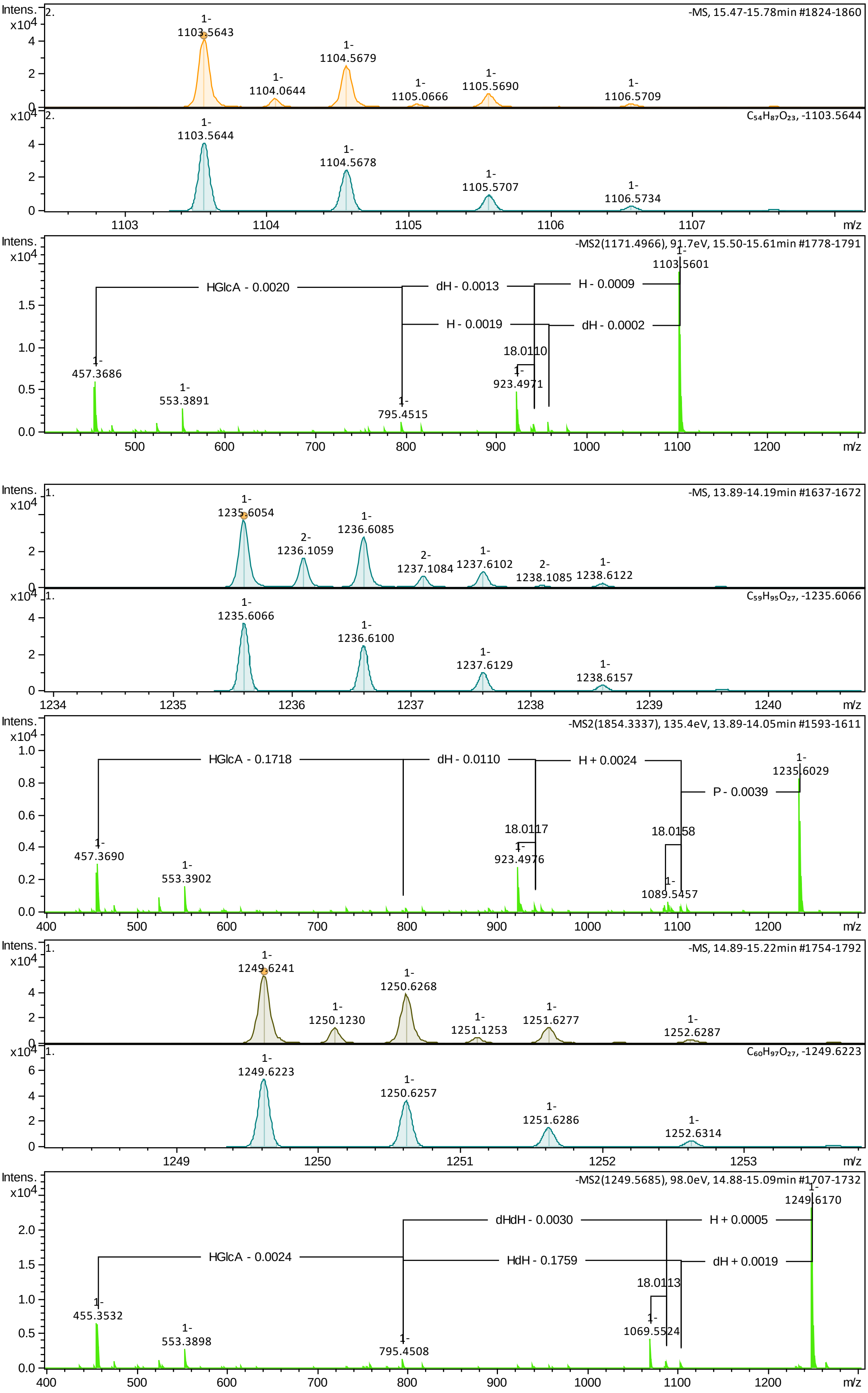

Figure S3: 1D and 2D NMR spectra of **PSI** (^{1}H, ^{13}C, COSY, HSQC).

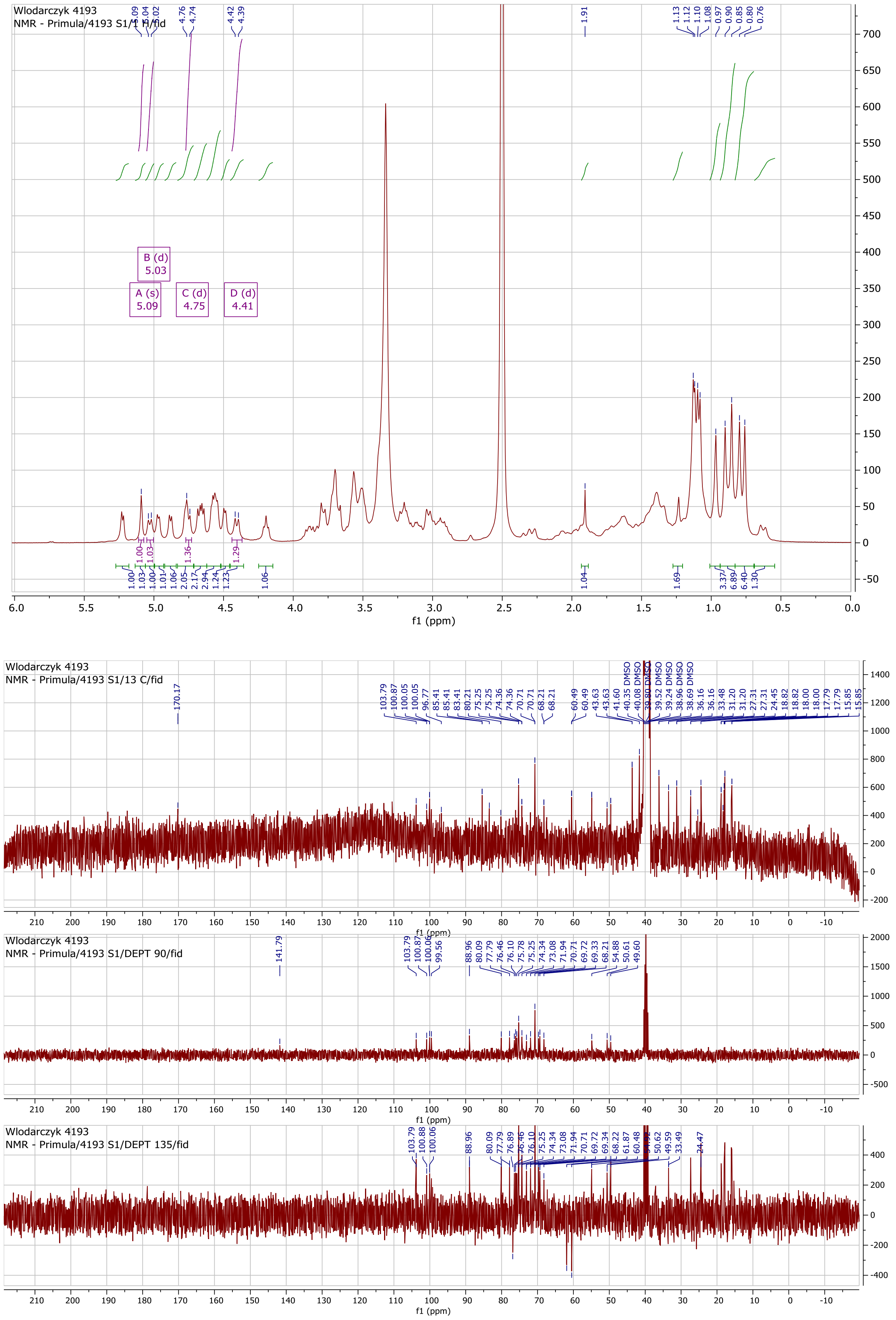

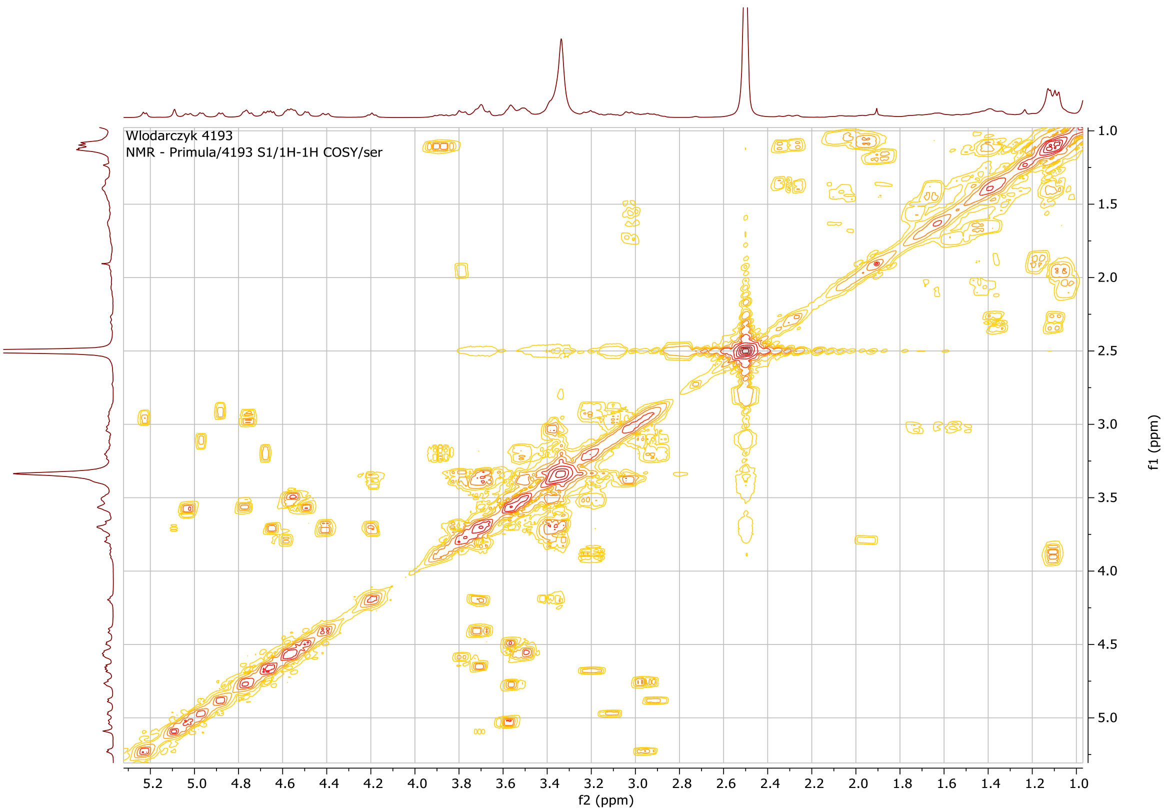

Wlodarczyk 4193
NMR - Primula/4193 S1/1H-1H COSY/ser
f2 (ppm)
f1 (ppm)

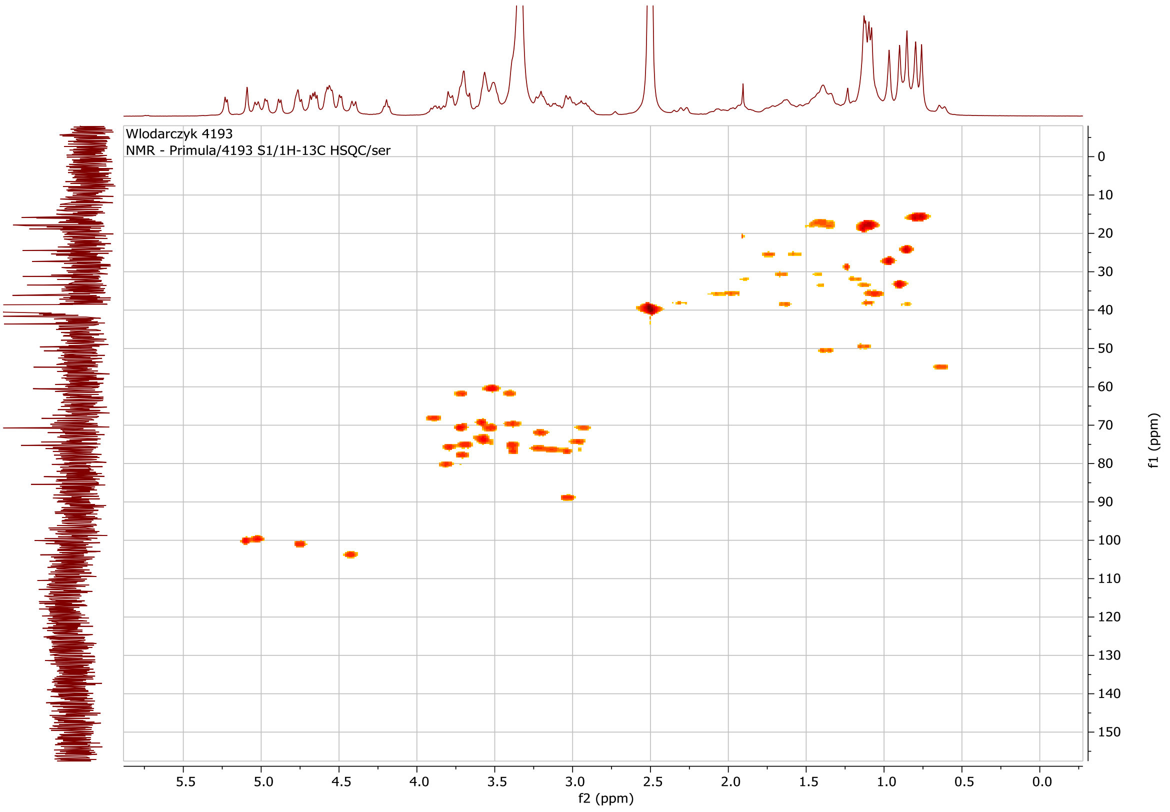

Wlodarczyk 4193
NMR - Primula/4193 S1/1H-13C HSQC/ser
f2 (ppm)
f1 (ppm)

Figure S4: 1D and 2D NMR spectra of **PSII** (^{1}H, ^{13}C, COSY, HSQC).

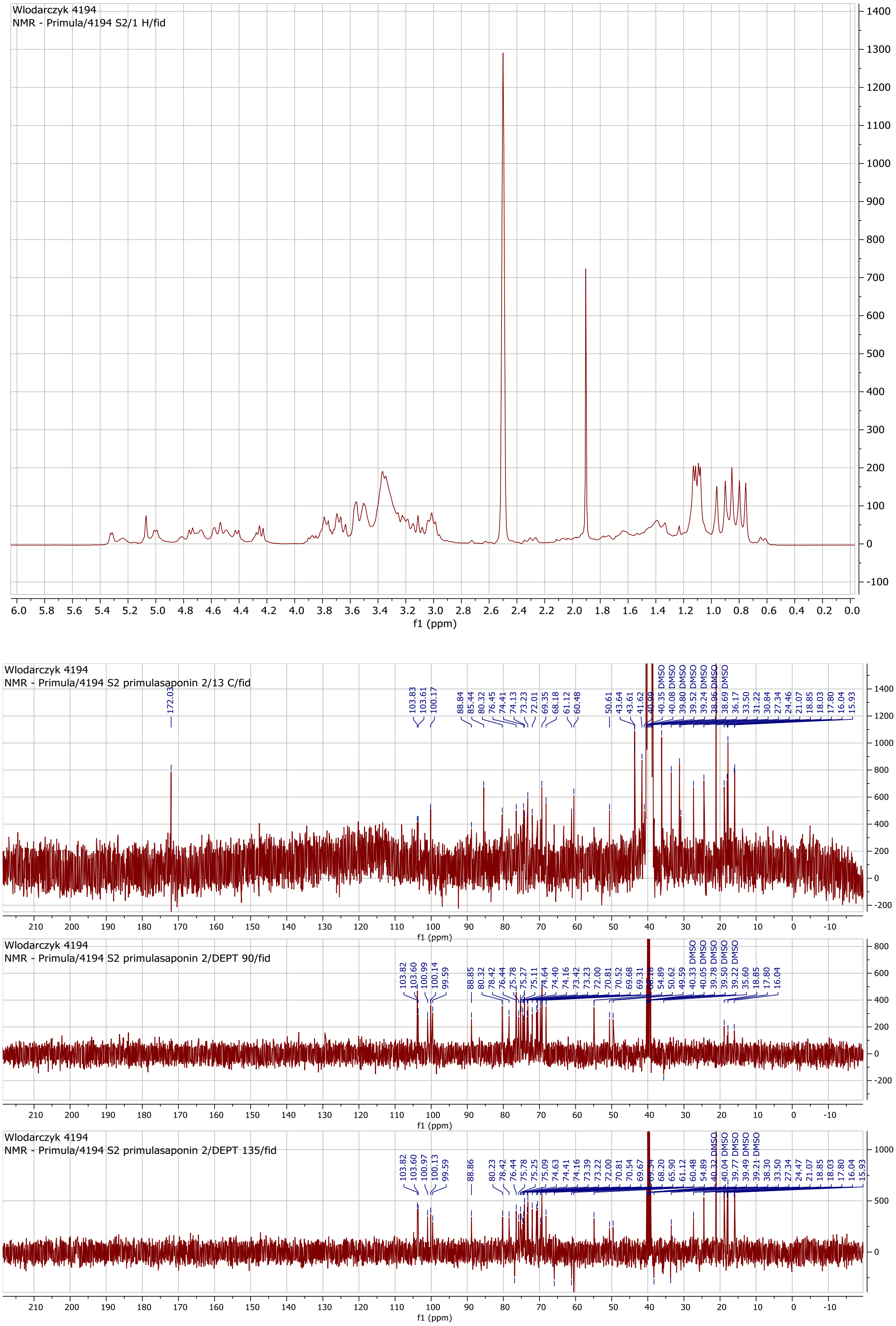

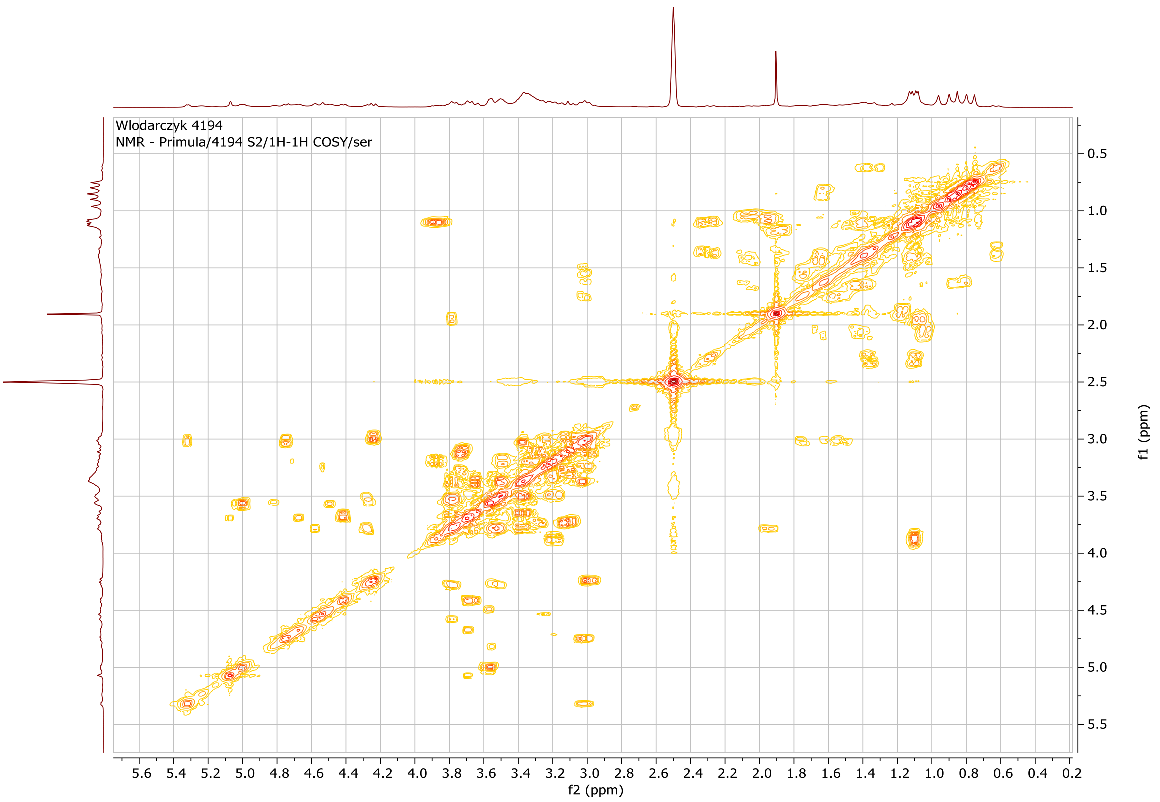
Wlodarczyk 4194
NMR - Primula/4194 S2/1H-1H COSY/ser
f2 (ppm)
f1 (ppm)

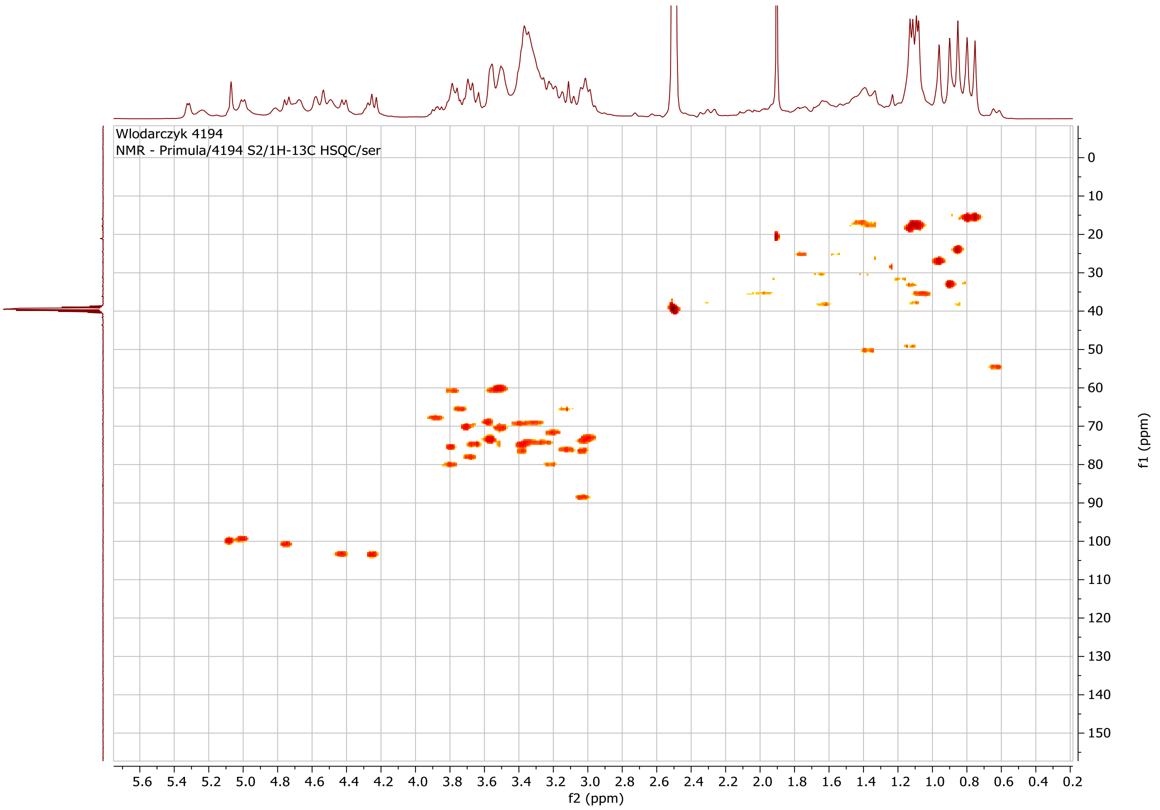
Wlodarczyk 4194
NMR - Primula/4194 S2/1H-13C HSQC/ser
f2 (ppm)
f1 (ppm)

Figure S5: 1D and 2D NMR spectra of **SSI** (^{1}H, ^{13}C, COSY, HSQC).

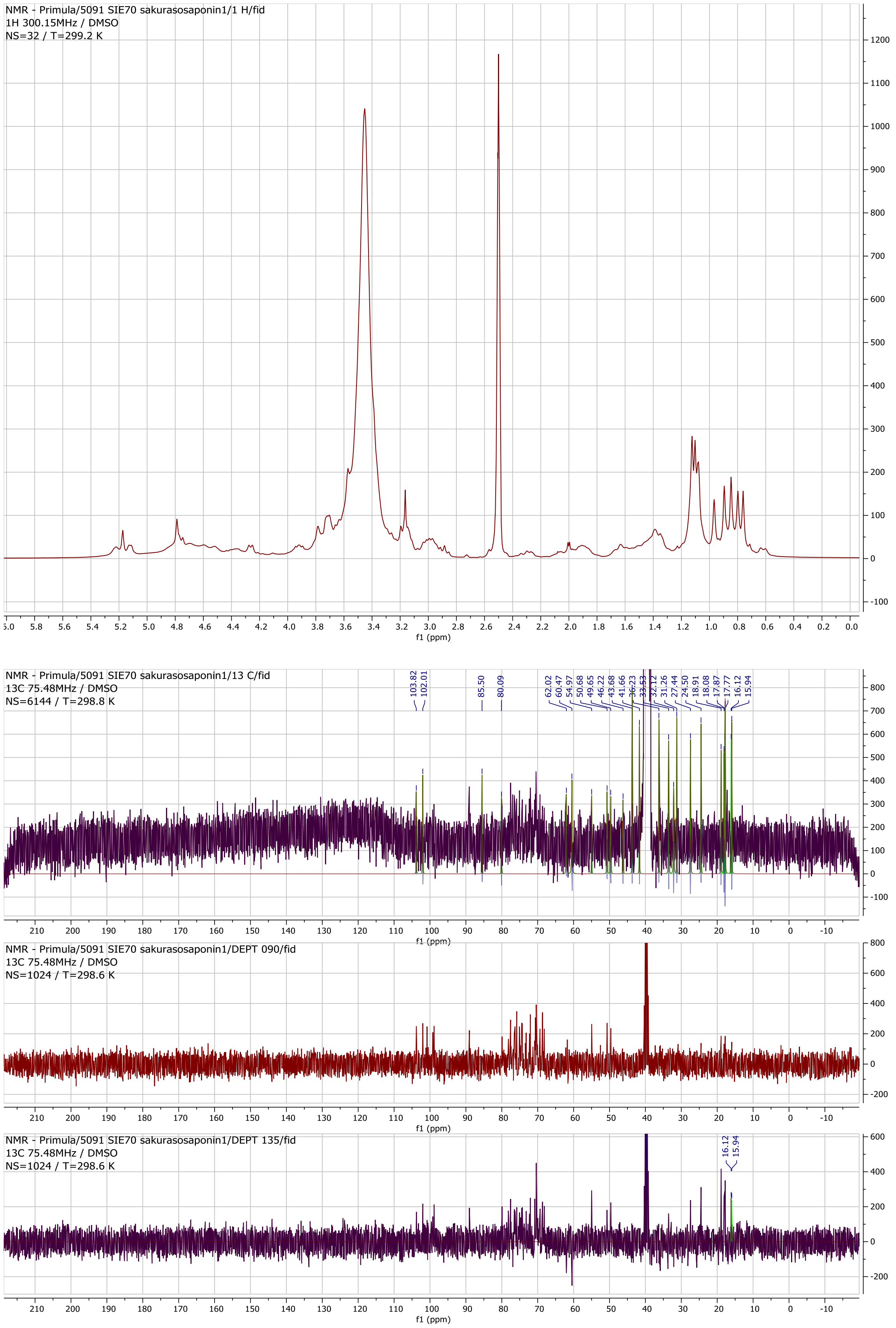

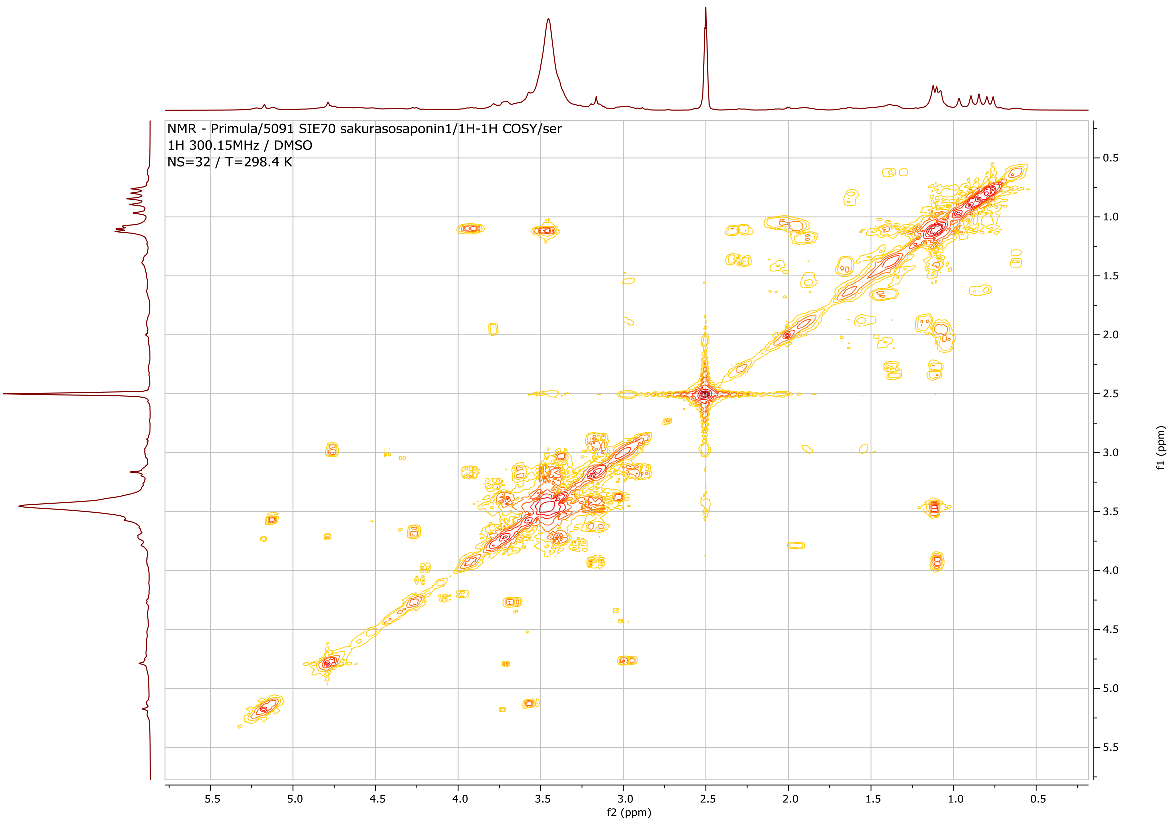
NMR - Primula/5091 SIE70 sakurasosaponin1/1H-1H COSY/ser
1H 300.15MHz / DMSO
NS=32 / T=298.4 K
f2 (ppm)
f1 (ppm)

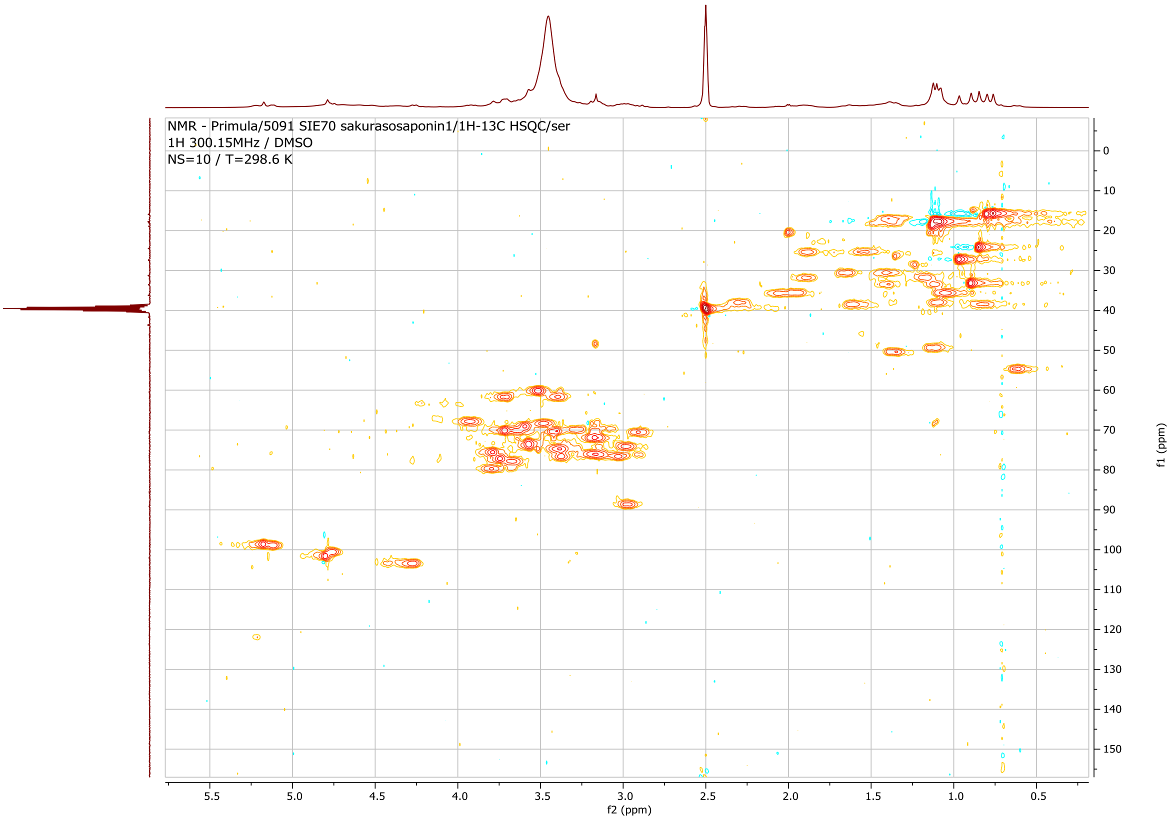
NMR - Primula/5091 SIE70 sakurasosaponin1/1H-13C HSQC/ser
1H 300.15MHz / DMSO
NS=10 / T=298.6 K
f2 (ppm)
f1 (ppm)

Figure S6: Purity determination protocol of compounds **PSI**, **PSII** and **SSI**.

EIT+	**SPRAWOZDANIE Z BADAŃ nr NMR-2017-05**<br>**z dnia 15.02.2017**	**Strona 2 z 7**<br>Egzemplarz nr ....

Identyfikacja wyposażenia pomiarowego (nazwa, typ)	NMR-BADD-000359-A1 (Spektrometr NMR700 MHz)
Badanie wykonał	Janusz Skonieczny

V. Wyniki

Kod próbki	Badany parametr	Metoda badawcza (nazwa, numer, data wydania)	Jednostka	Wynik
17-0165B	Czystość	Badanie czystości związków organicznych techniką NMR - badanie ilościowe, PB-NMR-02, edycja czwarta z dnia 04.11.2016	%	**88,91 (N)**
17-0166B				**78,14 (N)**
17-0167B				**91,89 (N)**

Badania oznaczone symbolem: A – badania akredytowane, zamieszczone w zakresie akredytacji PCA AB ......; lub N – badania nieakredytowane; lub P – badania akredytowane w Zakresie Akredytacji podwykonawcy (AB.......).

Laboratorium podaje niepewność: na życzenia Klienta, przy ocenie zgodności z wymaganiami, gdy jest istotne dla miarodajności wyników badań. W przypadku podawania niepewności, laboratorium określa niepewność jako niepewność rozszerzoną przy poziomie ufności 95% i współczynniku rozszerzenia k=2. Wynikom poniżej (<) powyżej (>) zakresu akredytacyjnego laboratorium nie podaje niepewności.
Wyniki badań i związana z nimi niepewność pomiaru nie obejmują etapu pobierania próbki.
Wyniki ze znakiem < lub> oznaczają, że wyniki są poniżej lub powyżej zakresu akredytacji i nie są akredytowane.
Laboratorium nie ponosi odpowiedzialności za pobranie i transport próbek jeśli próbki pobrał i dostarczył klient.
Wyniki odnoszą się wyłącznie do badanej próbki/ek.
Sprawozdanie zawiera 3 wynik/-ów badań i bez pisemnej zgody laboratorium nie może być powielane inaczej, jak tylko w całości.
Termin zgłaszania skarg nie może przekraczać 2 tygodni od daty wysłania Sprawozdania z Badań. Skargi można kierować na adres: jakosc@eitplus.pl

Dodatkowe informacje*
*stosować jeżeli dotyczy

V.1. Opis metody badawczej:
Pomiar qNMR (ilościowy NMR) za pomocą Spektroskopii Magnetycznego Rezonansu Jądrowego (NMR) z wykorzystaniem rezonansu jąder ^{1}H.
V.2. Dodatkowe informacje o wyposażeniu pomiarowym:
Pomiary wykonano na sondzie TXI (CP) w temperaturze 300K.
V.3. Przygotowanie próbek:
Użyto 5 mm probówek NMR, w których umieszczono po 600 µL roztworów badanych substancji i wzorców integracyjnych:
a.1. 1,440 mg PS I (17-0165B) i 1,092 mg NMR-D-12/04 w 1 mL deuterowanego metanolu CD_3OD (roztwór 1a).
a.2. 1,408 mg PS I (17-0165B) i 0,856 mg NMR-D-12/04 w 1 mL deuterowanego metanolu CD_3OD (roztwór 2a).
b.1. 3,328 mg PS II (17-0166B) i 0,752 mg NMR-D-12/04 w 1 mL deuterowanego metanolu CD_3OD (roztwór 1b).
b.2. 5,259 mg PS II (17-0166B) i 0,784 mg NMR-D-12/04 w 1 mL deuterowanego metanolu CD_3OD (roztwór 2b).

PO-15-Z-01, edycja 5 z dnia 20.05.2016r.

EIT+	**SPRAWOZDANIE Z BADAŃ nr NMR-2017-05**<br>**z dnia 15.02.2017**	
		**Strona 3 z 7**
		Egzemplarz nr ....

c.1. 1,987 mg SS I (17-0167B) i 0,898 mg NMR-D-12/04 w 1 mL deuterowanego metanolu CD_3OD (roztwór 1c).
c.2. 2,647 mg SS I (17-0167B) i 0,938 mg NMR-D-12/04 w 1 mL deuterowanego metanolu CD_3OD (roztwór 2c).
V.4. Wzorce integracyjne:
NMR-D-12/04 - Kwas maleinowy (Fluka; CAS: 110-16-7; Nr katalogowy / nr serii: 92816 / BCBM8127V).
V.5. Wzór strukturalny wzorca integracyjnego:

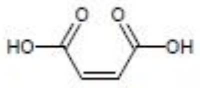

Kwas maleinowy (NMR-D-12/04)

V.6. Średnie masy molowe analizowanych saponin:
PS I: 1105.26 Da
PS II: 1237.38 Da
SS II: 1251.40 Da

V.7. Widma NMR:

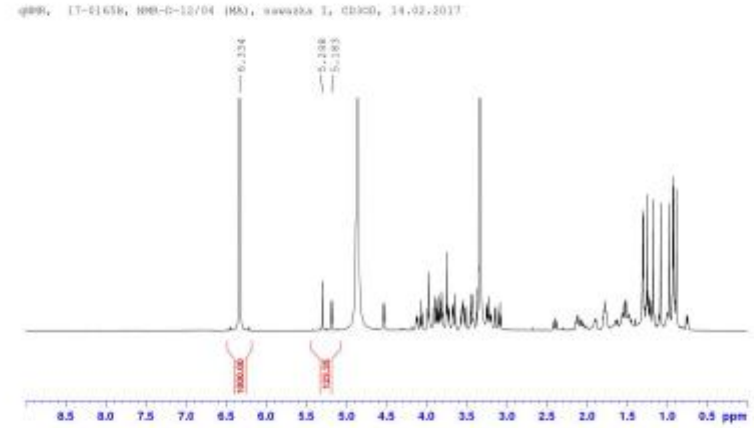

Widmo ^1H qNMR roztworu 1a.

EIT+	**SPRAWOZDANIE Z BADAŃ nr NMR-2017-05**<br>**z dnia 15.02.2017**	
		**Strona 4 z 7**
		Egzemplarz nr ....

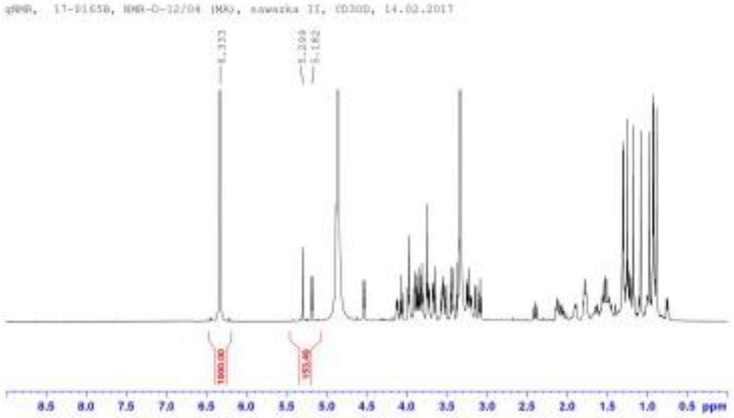

Widmo ^{1}H qNMR roztworu 2a.

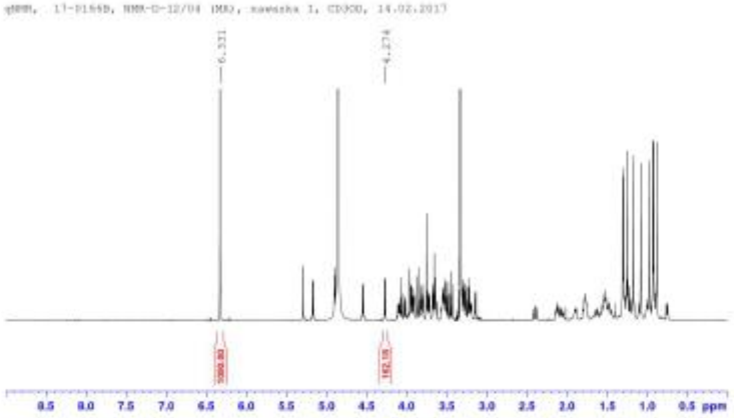

Widmo ^{1}H qNMR roztworu 1b.

EIT+	**SPRAWOZDANIE Z BADAŃ nr NMR-2017-05**<br>**z dnia 15.02.2017**	
		Strona 5 z 7
		Egzemplarz nr ....

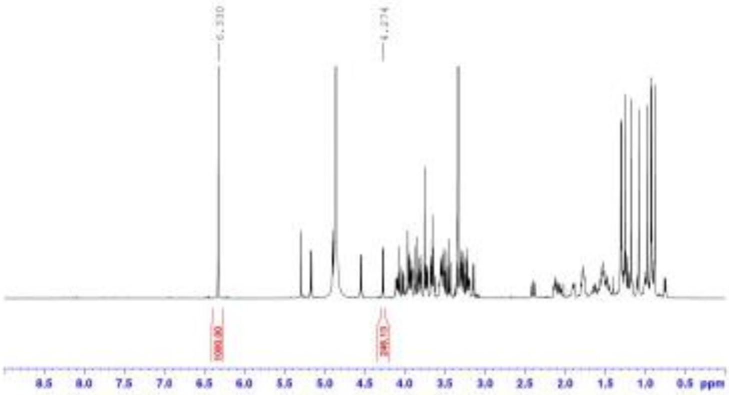

Widmo ^{1}H qNMR roztworu 2b.

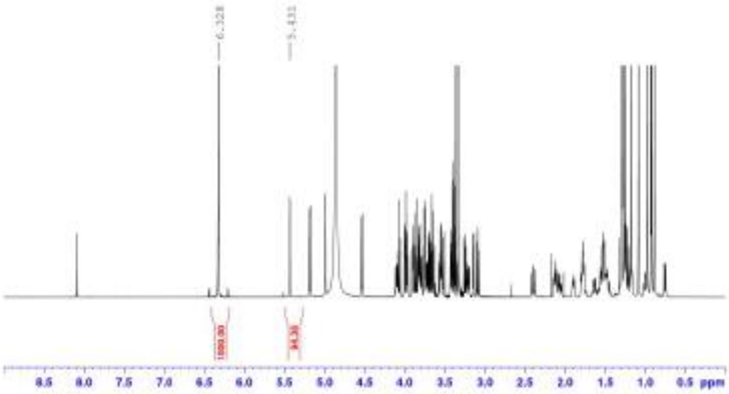

Widmo ^{1}H qNMR roztworu 1c.

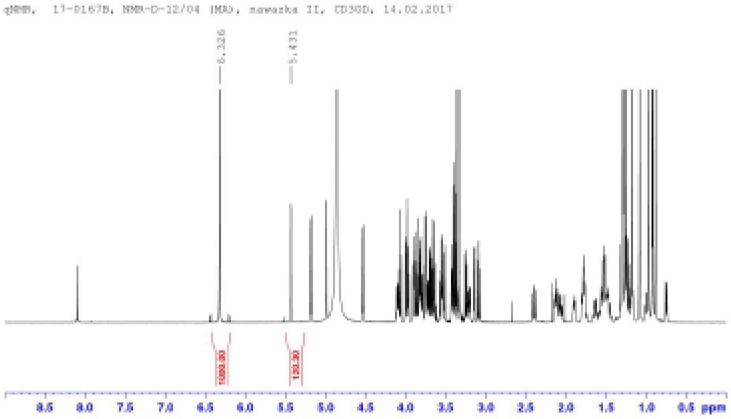

Widmo ^{1}H qNMR roztworu 2c.

V.8. Obliczenia:

Na podstawie poniższego wzoru wyznaczono czystość analitu:

$$P_x = \frac{I_x}{I_{Std}} \cdot \frac{N_{Std}}{N_x} \cdot \frac{M_x}{M_{Std}} \cdot \frac{m_{Std}}{m_x} \cdot P_{Std}$$

gdzie:

I_x – intensywności (pole powierzchni pod krzywą) wybranego sygnału analitu na widmie NMR

I_{std} – intensywności (pole powierzchni pod krzywą) wybranego sygnału wzorca na widmie NMR

N_x – liczba protonów biorących udział w rezonansie analitu

N_{Std} – liczba protonów biorących udział w rezonansie wzorca

M_x – masa molowa analitu

M_{Std} – masa molowa wzorca

m_x – masa naważki materiału badanego

m_{Std} – masa naważki wzorca

P_{Std} – czystość wzorca wewnętrznego

Czystość podano jako średnią z dwóch pomiarów spełniających kryteria akceptacji wyniku.

VI. Opinie i interpretacje*

*Stosować wówczas, gdy jest to uzasadnione merytorycznie lub wymagane przez Klienta (zgodnie z warunkami w zakresie akredytacji)

VII. Uwagi, załączniki

Sprawozdanie sporządzono w 2 jednobrzmiących egzemplarzach

Table S1: ^{1}H and ^{13}C NMR data of **PSI**, **PSII** and **SSI**. 300 MHz and 75 MHz respectively, DMSO-*d6*.

**standard**	**PSI**		**PSI**		**PSI**	
**position**	**δ_C (ppm)**	**δ_H (ppm)**	**δ_C (ppm)**	**δ_H (ppm)**	**δ_C (ppm)**	**δ_H (ppm)**
**1**	38.60 (t)	1.64 (m, 1H), 0.84 (m, 1H)	38.60 (t)	1.64 (m, 1H), 0.85 (m, 1H)	38.62 (t)	1.61 (m, 1H), 0.83 (m, 1H)
**2**	25.34 (t)	1.78 (m, 1H), 1.58 (m, 1H)	25.34 (t)	1.75 (m, 1H), 1.57 (m, 1H)	25.69 (t)	1.88 (m, 1H), 1.54 (m, 1H)
**3**	88.97 (d)	3.03 (dd, 1H)	88.84 (d)	3.02 (dd, 1H)	89.06 (d)	2.96 (dd, 1H)
**4**	39.00 (s)	-	38.90 (s)	-	39.05 (s)	-
**5**	54.96 (d)	0.63 (dd, 1H)	54.91 (d)	0.63 (dd, 1H)	54.97 (d)	0.61 (dd, 1H)
**6**	17.27 (t)	1.39 (m, 2H)	17.30 (t)	1.42 (m, 2H)	17.22 (t)	1.39 (m, 2H)
**7**	33.70 (t)	1.42 (m, 1H), 1.12 (m, 1H)	33.68 (t)	1.41 (m, 1H), 1.12 (m, 1H)	33.72 (t)	1.39 (m, 1H), 1.12 (m, 1H)
**8**	41.60 (s)	-	41.62 (s)	-	41.66 (s)	-
**9**	49.65 (d)	1.12 (m, 1H)	49.60 (d)	1.13 (m, 1H)	49.64 (d)	1.13 (m, 1H)
**10**	36.16 (s)	-	36.17 (s)	-	36.23 (s)	-
**11**	18.31 (t)	1.35 (m, 2H)	17.96 (t)	1.35 (m, 2H)	18.00 (t)	1.37 (m, 2H)
**12**	32.01 (t)	1.89 (m, 1H), 1.18 (m, 1H)	32.02 (t)	1.90 (m, 1H), 1.18 (m, 1H)	32.12 (t)	1.90 (m, 1H), 1.16 (m, 1H)
**13**	85.41 (s)	-	85.34 (s)	-	85.50 (s)	-
**14**	43.68* (s)	-	43.64* (s)	-	43.68* (s)	-
**15**	35.96 (t)	2.07 (m, 1H), 1.05 (m, 1H)	35.97 (t)	2.06 (m, 1H), 1.06 (m, 1H)	35.96 (t)	2.07 (m, 1H), 1.06 (m, 1H)
**16**	75.84 (d)	3.79 (m, 1H)	75.78 (d)	3.79 (m, 1H)	75.87 (d)	3.79 (m, 1H)
**17**	43.68* (s)	-	43.61* (s)	-	43.68* (s)	-
**18**	50.61 (d)	1.37 (m, 1H)	50.61 (d)	1.37 (m, 1H)	50.68 (d)	1.37 (m, 1H)
**19**	38.26 (t)	2.31 (m, 1H), 1.09 (m, 1H)	38.22 (t)	2.30 (m, 1H), 1.10 (m, 1H)	38.23 (t)	2.31 (m, 1H), 1.10 (m, 1H)
**20**	31.20 (s)	-	31.22 (s)	-	31.26 (s)	-
**21**	35.90 (t)	1.98 (m, 1H), 1.05 (m, 1H)	35.76 (t)	1.97 (m, 1H), 1.06 (m, 1H)	35.85 (t)	1.98 (m, 1H), 1.05 (m, 1H)
**22**	30.81 (t)	1.65 (m, 1H), 1.42 (m, 1H)	30.84 (t)	1.66 (m, 1H), 1.40 (m, 1H)	30.82 (t)	1.64 (m, 1H), 1.41 (m, 1H)
**23**	27.31 (q)	0.96 (s, 3H)	27.34 (q)	0.96 (s, 3H)	27.44 (q)	0.97 (s, 3H)
**24**	15.84 (q)	0.76 (s, 3H)	15.93 (q)	0.75 (s, 3H)	15.94 (q)	0.76 (s, 3H)
**25**	16.02 (q)	0.80 (s, 3H)	16.04 (q)	0.80 (s, 3H)	16.12 (q)	0.80 (s, 3H)
**26**	18.00 (q)	1.08 (s, 1H)	18.03 (q)	1.09 (s, 1H)	18.08 (q)	1.09 (s, 1H)
**27**	18.82 (q)	1.13 (s, 1H)	18.85 (q)	1.13 (s, 1H)	18.91 (q)	1.13 (s, 1H)
**28**	76.89 (t)	3.38 (m, 1H), 3.03 (m, 1H)	76.45 (t)	3.37 (m, 1H), 3.02 (m, 1H)	76.98 (t)	3.37 (m, 1H), 3.03 (m, 1H)
**29**	33.48 (q)	0.90 (s, 3H)	33.50 (q)	0.90 (s, 3H)	33.53 (q)	0.90 (s, 3H)
**30**	24.45 (q)	0.85 (s, 3H)	24.46 (q)	0.85 (s, 3H)	24.50 (q)	0.85 (s, 3H)
**GlcA'1(→3)**	103.79 (d)	4.42 (d, *J*=7.1 Hz, 1H)	103.83 (d)	4.42 (d, *J*=7.1 Hz, 1H)	103.82 (d)	4.27 (d, *J*=6.9 Hz, 1H)
**GlcA'6**	170.17 (s)	-	172.03 (s)	-	170.46 (s)	-
**Glc''1(→GlcA'3)**	100.87 (d)	4.74 (d, *J*=6.8 Hz, 1H)	100.99 (d)	4.75 (d, *J*=7.4 Hz, 1H)	100.81 (d)	4.76 (d, *J*=8.0 Hz, 1H)
**Glc''6**	61.87 (t)	3.70 (m, 1H), 3.40 (m, 1H)	61.12 (t)	3.80 (m, 1H), 3.56 (m, 1H)	62.02 (t)	3.71 (m, 1H), 3.40 (m, 1H)
**Xyl'''1(→Glc''4)**	-	-	103.61 (d)	4.26 (d, *J*=7.8 Hz, 1H)	-	-
**Xyl'''5**	-	-	65.90 (t)	3.72 (m, 1H), 3.12 (m, 1H)	-	-
**Gal''1(→GlcA'4)**	99.55 (d)	5.02 (d, *J*=6.4 Hz, 1H)	99.38 (d)	5.00 (d, *J*=5.8 Hz, 1H)	99.19 (d)	5.12 (d, *J*=6.2 Hz, 1H)
**Gal''6**	60.49 (t)	3.51 (m, 2H)	60.48 (t)	3.51 (m, 2H)	60.47 (t)	3.51 (m, 2H)
**R'''1(→Gal''4)**	100.05 (d)	5.09 (d, *J*=1.0 Hz, 1H)	100.13 (d)	5.08 (d, *J*=1.0 Hz, 1H)	98.85 (d)	5.18 (d, *J*=1.0 Hz, 1H)
**Rha'''6**	17.79 (q)	1.11 (dd, *J*=6.4 Hz, 3H)	17.80 (q)	1.10 (dd, *J*=6.2 Hz, 3H)	17.87 (q)	1.11 (dd, *J*=6.3 Hz, 3H)
**R''''1(→Rha'''2)**	-		-		102.01 (d)	4.79 (d, *J*=1.0 Hz, 1H)
**Rha''''6**	-		-		17.77 (q)	1.11 (dd, *J*=6.3 Hz, 3H)
